# Heavy metal removal applications using adsorptive membranes

**DOI:** 10.1186/s40580-020-00245-4

**Published:** 2020-11-16

**Authors:** Thi Sinh Vo, Muhammad Mohsin Hossain, Hyung Mo Jeong, Kyunghoon Kim

**Affiliations:** School of Mechanical Engineering, Sungkyunkwan University, Suwon, 16419 Republic of Korea

**Keywords:** Adsorption, Adsorptive membrane, Polymeric membrane, Polymer-ceramic membrane, Electrospinning membrane, Nano-enhanced membrane, Heavy metal removal

## Abstract

Water is a significant natural resource for humans. As such, wastewater containing heavy metals is seen as a grave problem for the environment. Currently, adsorption is one of the common methods used for both water purification and wastewater treatment. Adsorption relies on the physical and chemical interactions between heavy metal ions and adsorbents. Adsorptive membranes (AMs) have demonstrated high effectiveness in heavy metal removal from wastewater owing to their exclusive structural properties. This article examines the applications of adsorptive membranes such as polymeric membranes (PMs), polymer-ceramic membranes (PCMs), electrospinning nanofiber membranes (ENMs), and nano-enhanced membranes (NEMs), which demonstrate high selectivity and adsorption capacity for heavy metal ions, as well as both advantages and disadvantages of each one all, are summarized and compared shortly. Moreover, the general theories for both adsorption isotherms and adsorption kinetics are described briefly to comprehend the adsorption process. This work will be valuable to readers in understanding the current applications of various AMs and their mechanisms in heavy metal ion adsorption, as well as the recycling methods in heavy ions desorption process are summarized and described clearly. Besides, the influences of morphological and chemical structures of AMs are presented and described in detail as well.

## Introduction

Highly toxic and non-biodegradable types of heavy metal ions could result in grave health problems in both humans and animals [1, 2]. Therefore, heavy metal removal from wastewater is beginning to become a significant problem. Advancing technology developments has enabled heavy metal removal to be proposed through several efficient methods, namely, reverse osmosis, membrane filtration, ion exchange, electrochemical treatment, irradiation, extraction [3–8], etc. However, these methods have some disadvantages relating to high reagent requests, erratic heavy metal ion removal, toxic sludge creation, etc. In contrast, the adsorption method is concerned as a relatively simple method to design and conduct with economical, effective, and versatile properties [9]. Moreover, the results of the adsorption process by various investigators show the successful removal of heavy metals [10–12].

For adsorbents, in addition to low-cost adsorbents (i.e.: natural materials, bio-materials, and waste materials, etc.), membranes can also be concerned as effective adsorbents to be named adsorptive membrane (AM) owing to specific adsorption groups and exclusive morphological properties on the membranes to contribute support adsorption removal of heavy metal ions from wastewater. AMs are a truly good candidate for environmental protection by the purification of wastewater through the adsorption process. Considering this positive impact, herein, AMs are presented in this review article consisting of polymeric membranes (PMs), polymer-ceramic membranes (PCMs), electrospinning nanofiber membranes (ENMs), and nano-enhanced membranes (NEMs); moreover, advantages and disadvantages of different AMs fabrication methods all are shortly compared in Table 1. However, to evaluate the quality of the AMs for practical use, the avail oneself of these AMs have to be stable in chemical factor and reusable. Therefore, the regeneration and reuse of adsorbents are also one of the needful benefits that make this process more economical and environmentally friendly.

**Table 1:** Advantages and disadvantages of different AMs fabrication methods

AMs	Advantages	Disadvantages
PMs	Lots of selections for polymer material Easy to incorporate polymer materials together Membranes with smooth/porous surface membrane Applying for regeneration and reuse	Be limited to thermal stability
PCMs	Simple and rough fabrication method Lamellar structure: non-toxicity, low-cost, high cation exchangeability, and mechanical and chemical stabilities	Foul, slower, and more extreme recovery methods Lots of depressions and microcracks on the membrane surface due to the manual compaction and deformation during the ceramics firing process Unreachable sites and low surface areas due to the stack of lamellar structures Be limited about recycling number
ENMs	Lots of selections for the material Easy to incorporate additives in nanofibers High versatility in control of nanofiber diameter, microstructure, and arrangement Membranes with high porosity (> 90.0%) and high surface-to-volume ratio Abundant nanostructures: bilayer, tri-layer nanofibers Applying for regeneration and reuse	Difficult to attain nanofibers with diameters below 100 nm Difficult to attain ENMs with maximum pore sizes smaller than 100 nm Slow yield speed
NEMs	Larger surface contact, higher reactivity, and better disposal ability Best describing the function of the nanomaterials in the membrane High aspect ratio, mechanical strength, compatibility of the carbon matrix with the polymeric structure, and strong interactions and adhesion Applying for regeneration and reuse	Requires particles with narrow size distribution Decreasing energy demand Need to use chemicals for membrane cleaning, membrane durability, and membrane performance

**Fig. 1:**
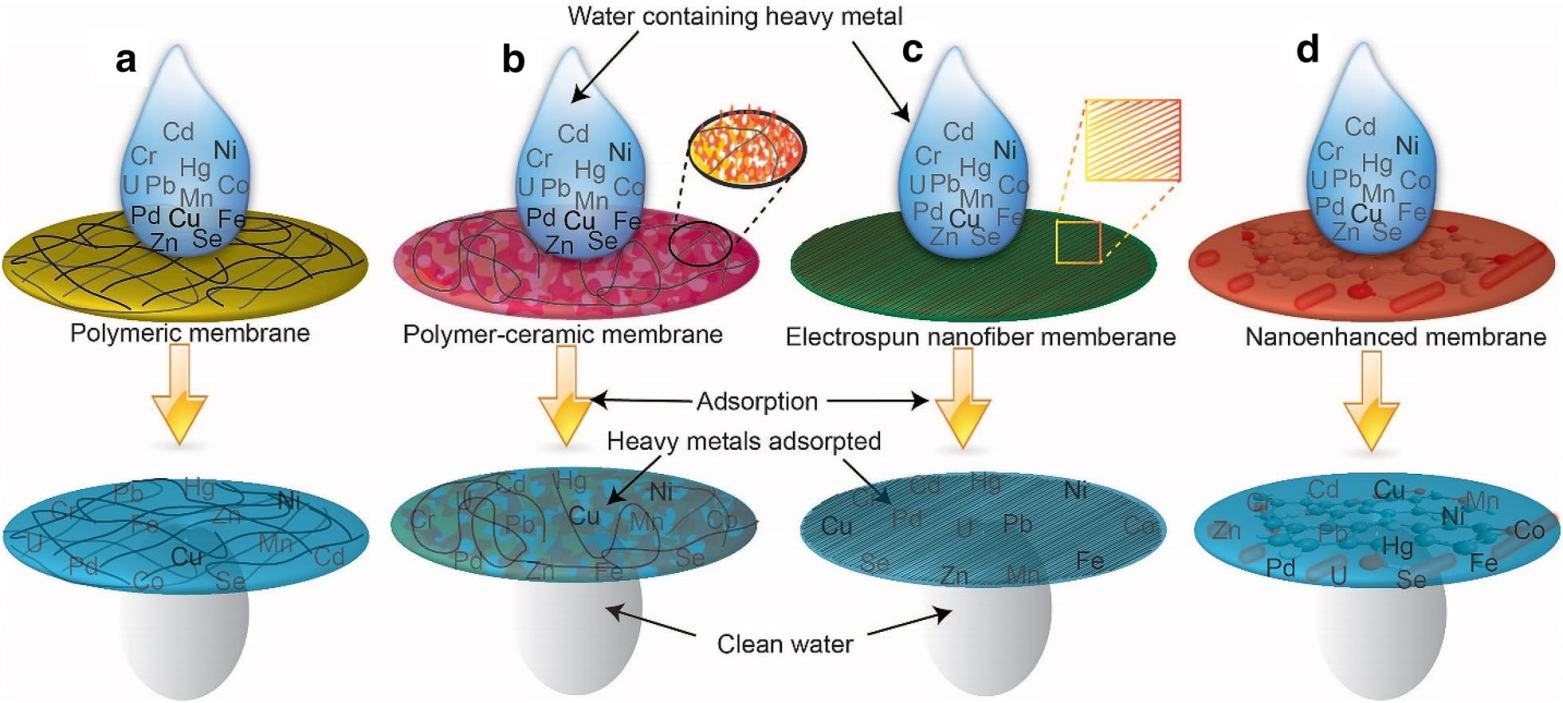
Scheme of different adsorptive membranes (AMs) in the removal of heavy metal. **a** polymeric membranes (PMs) are created from polymer source materials, **b** polymer-ceramic membranes (PCMs) are created from a combination between polymeric and ceramic (natural clay materials: bentonite, kaolinite, and montmorillonite) materials, **c** electrospinning nanofiber membranes (ENMs) are created from electrospinning method for forming fibers with nanometer to micron diameters, and **d** and nano-enhanced membranes (NEMs) are created from incorporating nanomaterials (carbonaceous materials, nanometal or nanometal oxides, and other organics)

In this review article, the geometries of an AM, the general theory of adsorption of heavy metal on a membrane surface for both the adsorption isotherms and adsorption kinetics are introduced to support the readers further understanding. The AMs (Fig. 1) include PMs, PCMs, ENMs, and NEMs to be summarized and described in detail the influences of their morphological and chemical structures. Especially, their applications and recycling performances are summarized and compared in short to support the reader's understanding of a clear overview of it. Besides, the recently advanced adsorbents from nanoparticles were used as fillers in the membrane to enhance its performance are presented as well.

## Adsorption conceptions on AMs surface

### General theories

**Fig. 2:**
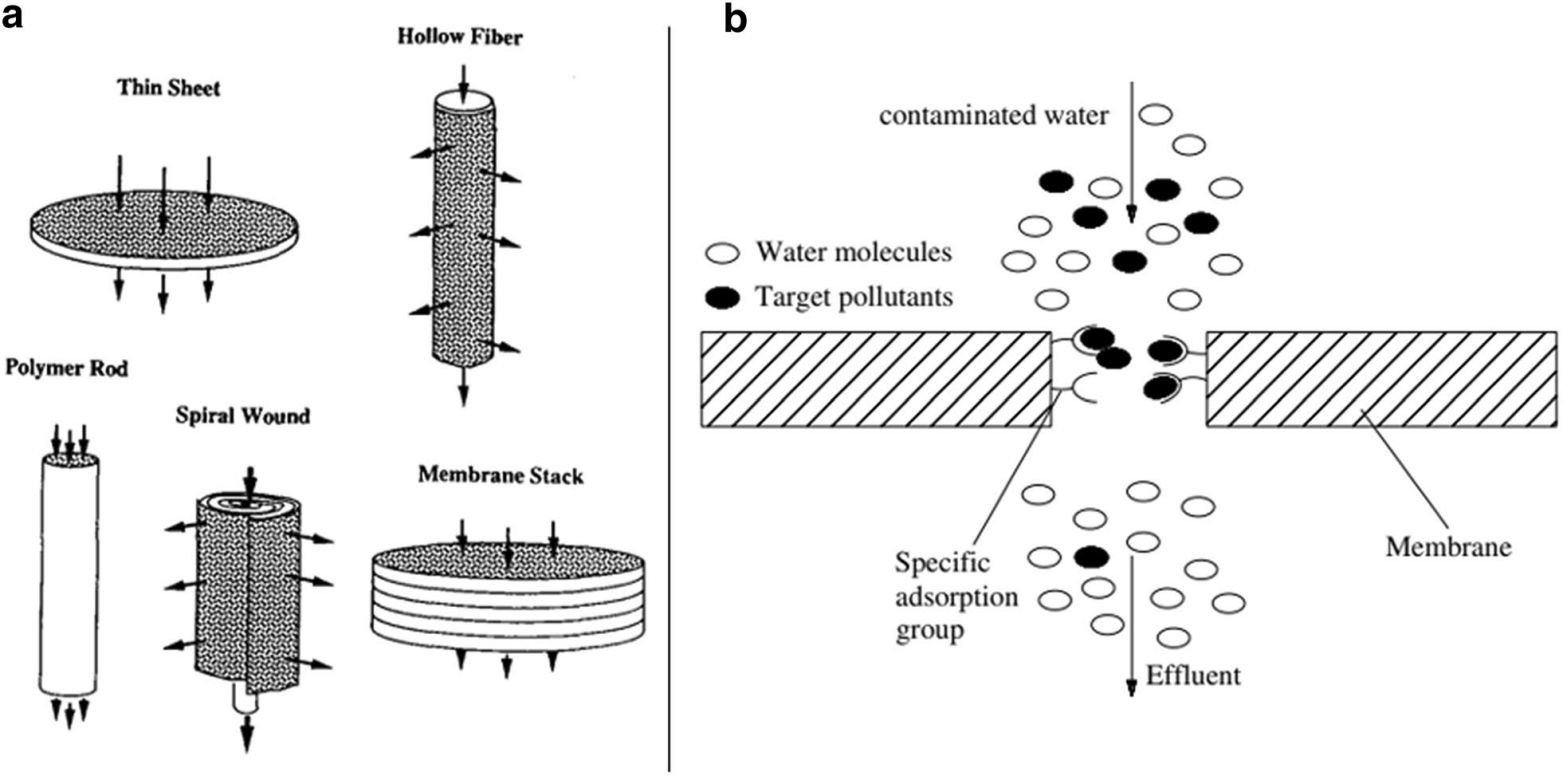
**a** Scheme of AMs and arrows note for the directions of bulk flow (Reprinted from [13]), and **b** the principle of AMs (Reprinted from [14])

AMs are available with a count of commercial geometries or prepared geometries from the laboratory (Fig. 2a) [13, 14]. Remarkably, the single AMs with thin sheets and hollow fibers are versatile, inexpensive, convenient, rapidly adsorb at low-pressure, and continuously recycle. Additionally, these single AMs are stacked in series and housed in a rigid cylindrical shell to achieve the necessary adsorption capacities due to the limited recovery ability of the single AMs, which are named spiral wound and membrane stack. For both spiral-wound and membrane stack, there are several advantages including high compatible ability, and the cross-sectional dimension is perpendicular to the direction of the flow significantly longer than the flow path, as well as resistant settling and cracking with keeping frictional support in the column wall. Hence, these membranes-based columns residence times are shorter and backpressures are smaller than those of the single AMs with the large volumetric capacity in the large-scale.

In 1881, the word "adsorption" was coined by German physicist Heinrich Kayser (1853–1940) [15]. AM technology was expanded in the mid-1980s [16, 17]. Theoretically, adsorption is seen as a transfer progression of mass, which means that a transferred substance from the liquid phase to a solid surface is passed through physical and/or chemical interactions. First, physical adsorption (or physisorption) is based on the van der Waals force, which is attributable to the electronic structure of the upset atom (or molecule) upon adsorption [18]. Next, the chemical adsorption (or chemisorption) primarily relates to a chemical reaction of the adsorbent surfaces [19]. The connection of significant functional groups on the surfaces and pore walls of membranes is the basis upon which AMs are built to selectively adsorb pollutants (Fig. 2b) [14]. Specifically, the significant functional active sites could attach with the pollutants to allow contaminant removal from water with high adsorption capacity during exudation of wastewater across the membrane [14]. Generally, the physical adsorption needs a short interval of contact time for the adsorbed molecules, it is the opposite for the chemical adsorption due to strong chemical bonding of the adsorbent with adsorbate to achieve the attainment of equilibrium with longer contact time.

Overall, the process of heavy metal removal is occurred on the solid surface by adsorption, and the equilibrium is obtained by achieving constant concentrations of the adsorbed heavy metals in water. Stronger interactions in chemical adsorption are more common than those in physical adsorption for heavy metal removal to achieve higher adsorption capacity. Thus, the required criteria for AM-based adsorbents for removal of heavy metals consist of four major issues: (i) AMs are nontoxic and friendly; (ii) AMs offer high adsorption capacity to the low concentrations of pollutants; (iii) AMs enable removal of pollutants from their surfaces easily; (iv) AMs can be reused and recycled.

### Adsorption evaluation

In the adsorption process, the adsorption evaluation is based on the amount of adsorbate at equilibrium (q_e_; mg g^−1^). It is typically computed with the balance of material during an adsorption process, the disappeared adsorbate from the solution, and the adsorbent mass [20] [Eq. (1)].

1 $${\mathrm{q}}_{\mathrm{e}}= \frac{{\mathrm{C}}_{\mathrm{o}}-{\mathrm{C}}_{\mathrm{e}}}{\mathrm{m}}*\mathrm{V}$$

2 $$\mathrm{R }(\mathrm{\%})= \frac{{\mathrm{C}}_{\mathrm{o}}-{\mathrm{C}}_{\mathrm{e}}}{{\mathrm{C}}_{\mathrm{o}}}*100$$

where C_o_ (mg  L^−1^) is the initial adsorbate concentration, C_e_ (mg L^−1^) is the equilibrium adsorbate concentration in the solution, V (L) is a volume of the adsorbate solution, and m (g) is the dried mass of employed adsorbent. The adsorption evaluation is referred to as the adsorbate removal efficiency (R, %) from the solution [21], as shown in Eq. (2).

### Adsorption isotherms

The relationship between the amounts of adsorbed heavy metal ions in water is termed an adsorption isotherm [22] at equilibrium and could be based on Langmuir and Freundlich models. In the Langmuir model, adsorption occurs uniformly on the active sites of the adsorbent. Once adsorbates occupy the active sites, the adsorption is naturally terminated at this site. In the non-linear Langmuir model, its equation is presented in Eq. (3) [23, 24], where q_max_ is the maximum adsorption capacity (mg g^−1^) of adsorbent, K_L_ is the equilibrium constant (Lmg^−1^), C is the equilibrium concentration (mgL^−1^), and q is the amount of adsorbed metals at equilibrium (mg g^−1^). Moreover, the linear Langmuir model is stated in Eq. (4), where q_m_ is the saturated monolayer adsorption capacity, and b is the adsorption equilibrium constant. The plot of C_e_/q_e_ vs. C_e_ provides a straight line. The maximum adsorption capacity and the bond energy of adsorbates are determined with the slope and intercept. In the Freundlich model, its equation is seen as an experienced model of multilayer adsorption on the adsorbent. The non-linear Freundlich model is shown in Eq. (5) [25]. The linear Freundlich model is described in Eq. (6), where q_e_ is a loading of adsorbate on adsorbent at equilibrium (mg g^−1^), K_F_ is an indicator of adsorption capacity (mg^1−n^ L^n^ g^−1^), n is adsorption energetics, and C_e_ is the aqueous concentration of adsorbate at equilibrium (mg L^−1^).

3 $$\mathrm{q}= \frac{{\mathrm{q}}_{\mathrm{max}}{\mathrm{K}}_{\mathrm{L}}\mathrm{C}}{1+ {\mathrm{K}}_{\mathrm{L}}\mathrm{C}}$$

4 $$\frac{{\mathrm{C}}_{\mathrm{e}}}{{\mathrm{q}}_{\mathrm{e}}}= \frac{{\mathrm{C}}_{\mathrm{e}}}{{\mathrm{q}}_{\mathrm{m}}}+ \frac{1}{{\mathrm{bq}}_{\mathrm{m}}}$$

5 $${\mathrm{q}}_{\mathrm{e}}= {\mathrm{K}}_{\mathrm{F}}{\mathrm{C}}_{\mathrm{e}}^{\mathrm{n}}$$

6 $${\mathrm{logq}}_{\mathrm{e}}=\mathrm{ log}{\mathrm{K}}_{\mathrm{F}}+ \frac{{\mathrm{logC}}_{\mathrm{e}}}{\mathrm{n}}$$

**Fig. 3:**
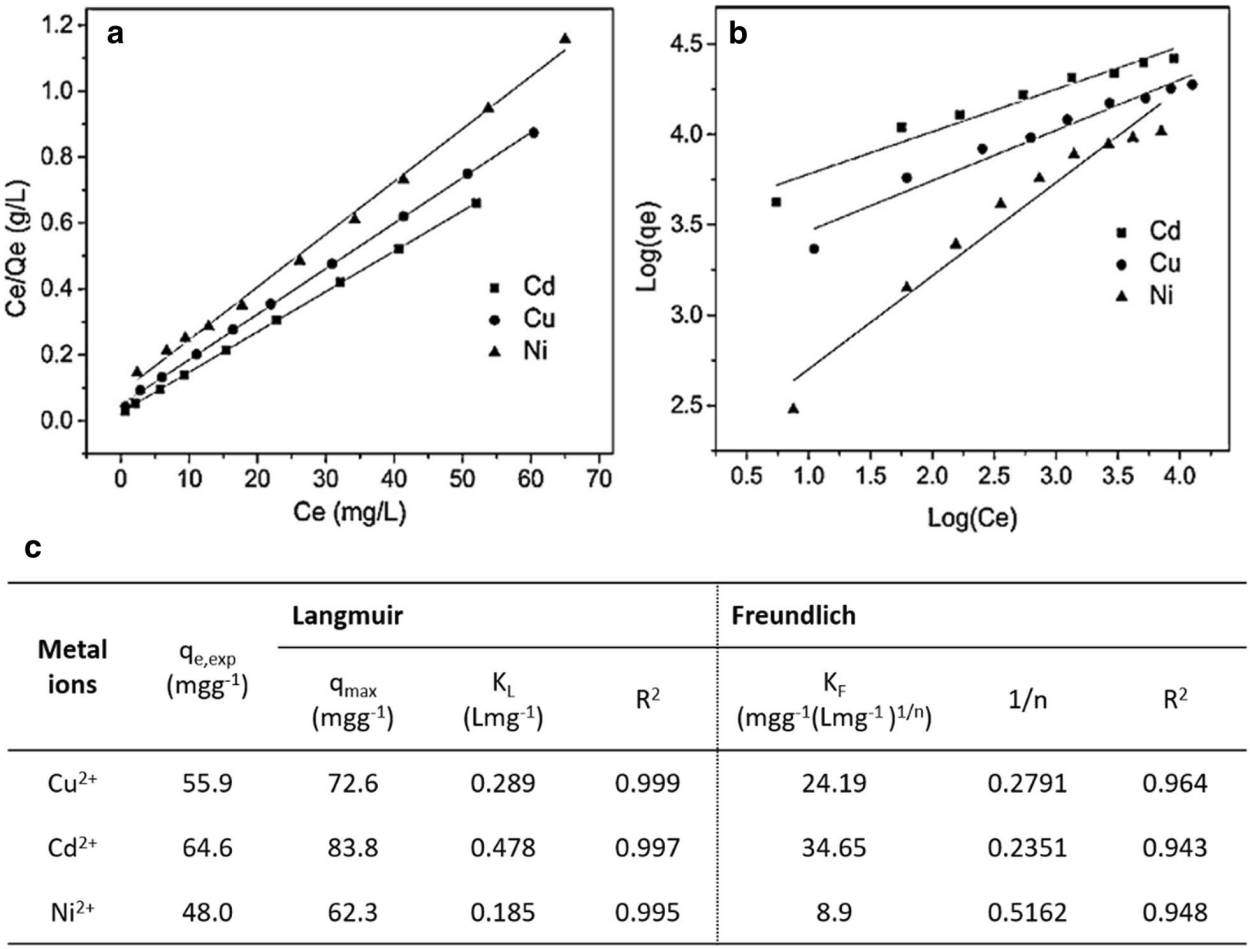
**a** Langmuir, **b** Freundlich isotherms, and **c** parameters of all models for the Cd^2+^, Cu^2+^, and Ni^2+^ ions adsorption on GO membranes. (Reprinted from [26])

In summary, the Langmuir model is considered as a simple theoretical adsorption isotherm, which is assumed on adsorption homogeneity, monolayer surface adsorption with a limited number of identical sites (i.e.: energy is equivalent in all sites), and no interaction between adsorbed molecules. Besides, the Freundlich model is seen as an empirical relationship, which is assumed on heterogeneous surface adsorption. It means that the adjacent sites are occupied or not when the attaching ion adsorption energy is onto an adsorption site of the adsorbate. Especially, this Freundlich isotherm could not predict the adsorption capacity of the adsorbents at equilibrium time. For instance, the Langmuir and Freundlich models for Cd^2+^, Cu^2+^, and Ni^2+^ ions adsorption on GO membranes [26] were shown in Fig. 3a, b, respectively. The calculated parameters of the above isotherm models were also listed in Fig. 3c. The Langmuir model was better fitted than that of the Freundlich model (R^2^—correlation coefficient ~ 0.999). It showed that the studied metal ions on the GO membrane were monolayer coverage, as well as 1/n constant of the Freundlich model was lower than 1 to be seen as a favorable process.

Additionally, both the above models could be combined, a new name is Langmuir–Freundlich model [27], which is assumed interaction between each adsorption site and only one adsorbate molecule. Furthermore, there are various well-known models used to interpret the results of adsorption processes [28–36] for the adsorption equilibrium between an adsorbate in solution and the surface of the adsorbent, such as, Redlich–Peterson model [30] is proposed as a three-adjustable-parameter semi-empirical isotherm to depict multilayer adsorption on both heterogeneous and homogeneous surfaces, which is used to consider the limitations of both the Langmuir and Freundlich models.

### Adsorption kinetics

Further understanding of the adsorption process can be achieved by defining and explaining the mechanisms involved in metal adsorption processes and the major parameters regulating adsorption kinetics. There are two common kinetic models that several scientists use, such as the pseudo-first-order model and the pseudo-second-order model. The pseudo-first-order model is seen as a kinetic model regularly used for the adsorption process, which is based on Lagergren’s pseudo-first-order expression, as shown in Eq. (7) [37]. In Eq. (7), q_e_ and q_t_ are the amounts of adsorbed metal ions per gram of adsorbents (mg g^−1^) at equilibrium and at time t (min), respectively, and k_1_ is the pseudo-first-order rate constant for the adsorption process (min^−1^). Alternatively, its linear form is presented in Eq. (8) and is derived by integrating this kinetic expression with the initial condition (q_t_). The plot of 1/q_t_ vs. 1/t is a straight line, and k_1_ is the rate constant of the pseudo-first-order related to the line’s slope. A pseudo-second-order model could be achieved based on Ho’s pseudo-second-order model, which is grounded on the adsorption hypothesis through second-order chemisorption. It is stated in the linear expression [38] (Eq. 9), where k_2_ is the pseudo-second-order rate constant.

7 $$\frac{{\mathrm{dq}}_{\mathrm{t}}}{\mathrm{dt}}={\mathrm{k}}_{1}({\mathrm{q}}_{\mathrm{e}}-{\mathrm{q}}_{\mathrm{t}})$$

8 $$\frac{1}{{\mathrm{q}}_{\mathrm{t}}}=\frac{1}{{\mathrm{q}}_{\mathrm{e}}}+\frac{{\mathrm{k}}_{1}}{{\mathrm{q}}_{\mathrm{e}}\mathrm{t}}$$

9 $$\frac{\mathrm{t}}{{\mathrm{q}}_{\mathrm{t}}}=\frac{1}{{\mathrm{k}}_{2}{\mathrm{q}}_{\mathrm{e}}^{2}}+\frac{\mathrm{t}}{{\mathrm{q}}_{\mathrm{e}}}$$

10 $${\mathrm{q}}_{\mathrm{t}}= {\mathrm{K}}_{\mathrm{id}} {\mathrm{t}}^{0.5}+{\mathrm{C}}_{\mathrm{i}}$$

**Fig. 4:**
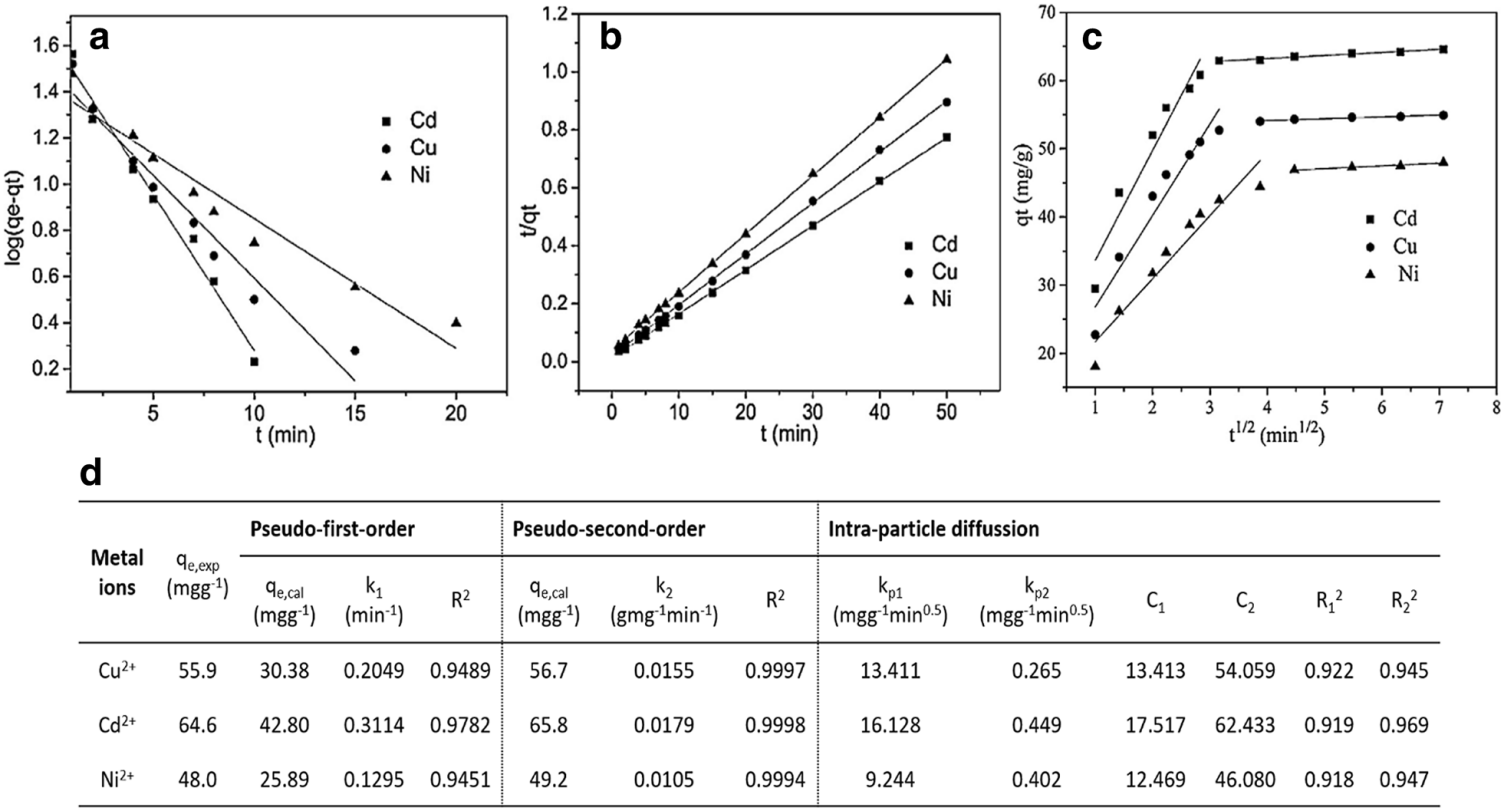
**a** Pseudo-first-order, **b** pseudo-second-order, **c** intra-particle diffusion models, and **d** parameters of all models for the Cd^2+^, Cu^2+^ and Ni^2+^ ions adsorption on GO membranes. (Reprinted from [26])

Also, other models are proposed by several researchers [39, 40] as well; in particular, Weber and Morris [41] (Eq. 10) proposed an intra-particle diffusion model, which is further employed to investigate the diffusion mechanism during adsorption, where K_id_ (mg g^−1^min^0.5^) is the rate constant of intra-particle diffusion, t^0.5^ is the square root of the time, and C_i_ is the intercept at stage i. For example, the pseudo-first-order and pseudo-second-order models for Cd^2+^, Cu^2+^, and Ni^2+^ ions adsorption on GO membranes [26] were shown in Fig. 4a, b respectively, the calculated parameters of the above kinetic models were also listed in Fig. 4d. The pseudo-second-order model was better fitted than that of the pseudo-first-order model (R^2^ ~ 0.9999) and the calculated q_e_ values of the pseudo-second-order model were close more to the experimental ones; therefore, the adsorption rates of these metal ions on GO membranes were assigned through chemical adsorption. Besides, the intra-particle diffusion model for Cd^2+^, Cu^2+^, and Ni^2+^ ions adsorption onto GO membranes was also used to express the relationship of two straight portions as showed in Fig. 4c [i.e.: (1) an external diffusion—an adsorbate diffusion from the solution to the adsorbent interface space; (2) an intra-particle diffusion—an adsorbate diffusion inside the adsorbent pore]. As listed in Fig. 4d, k_d1_ value (first intra-diffusion rate constant) of the first portion is higher than that (k_d2_—second intra-diffusion rate constant) of the second portion, as well as C_1,_ is lower than C_2_, suggesting that the studied metal ions removal rate was higher in the starting, then this adsorption rate was controlled through intra-particle diffusion when the adsorption ability obtained equilibrium, and the intra-particle diffusion was considered as part of the adsorption but was not the only rate-controlling stage.

In general, the studies on adsorption kinetics are truly necessary to predict optimal conditions in full-scale batch adsorption processes. Thus, kinetic models will provide information about adsorption mechanisms as well as rate-controlling steps (i.e.: chemical reaction or mass transport processes). They consist of pseudo-first-order, pseudo-second-order (non-linear or linear forms), intra-particle diffusion, etc. are employed to investigate the adsorption kinetics; in particular, the pseudo-first and the pseudo-second-order kinetic equations are used the most prevalently.

## Applications of AMs in heavy metal removal

### Applications of PMs

Adsorbents from polymer materials were expanded in the 1960s [42]. In the adsorption process, pollutants are adsorbed into the significant functional groups on AMs during a permeation of wastewater crossing the membrane. Significant functional groups such as amine, carboxyl, and sulfonic acid in synthesized polymers (or biopolymers), enable their adsorption capacities for heavy metal removal are effectual [43–45]. In particular, chitosan (CTS)-based AMs have been extensively used to remove various contaminants from wastewater, owing to the amino and hydroxyl groups on CTS. Hence, the metal ions adsorption on CTS could relate to different mechanisms including chelation, electrostatic attraction, ion exchange, etc., which depend on the pH values, the composition of the solution, and metal ions features. There are many studies on adsorption kinetics of CTS-based AMs that have been carried out for heavy metal removal from wastewater [46]. Moreover, CTS could influence the surface charge of AMs [47], which means their susceptibilities induce positive charges onto the other PMs. For instance, Reiad et al. [48] demonstrated the removal of Fe^2+^ and Mn^2+^ from aqueous by using CTS/poly(ethylene glycol) (CTS/PEG) membranes to the adsorption capacities of up to 38.0 mg g^−1^ and 18.0 mg g^−1^. This suggests that the increasing amount of CTS in the blend led to the rising adsorption capacity of metal ions, but its stability decreased. Moreover, the adsorption capacity of this membrane increased more for Fe^2+^. CTS-based AMs have studied for Cu^2+^ and Hg^2+^ adsorption by Vieira et al. [49].

**Fig. 5:**
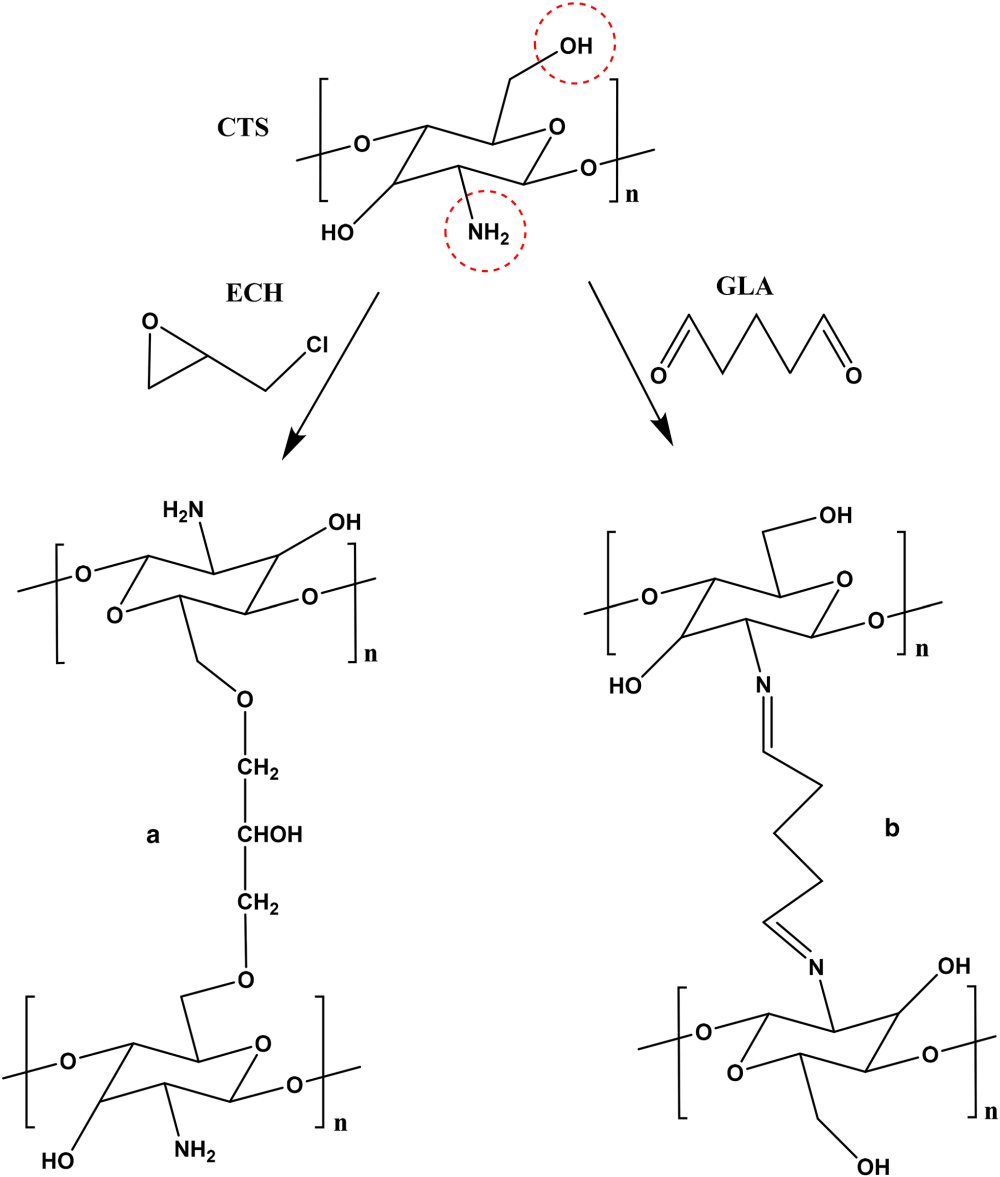
Formed structures of crosslinking of CTS using **a** ECH and **b** GLA

A cross-linking reaction could support increasing the adsorption capacity for metal ions [50–52]. The use of epichlorohydrin (ECH) or glutaraldehyde (GLA) in the crosslinking of CTS is generally performed to prevent the dissolution of CTS in acidic conditions or to appreciate the adsorption capacities for heavy metal. ECH binds to hydroxyl groups of CTS (Fig. 5a), and GLA is bonded to amino groups of CTS (Fig. 5b), which makes it practical to employ both crosslinking progressions to explain their adsorption mechanism. Comparing to natural CTS (nCTS), the crosslinking of CTS with ECH and GLA (ECH-cCTS and GLA-cCTS) has achieved adsorption capacities of Hg^2+^ higher than that of nCTS (Table 2) [50]. Besides, the maximum adsorption capacity of ECH-cCTS increased slightly comparing to nCTS (Table 2), suggesting that the interaction of Hg^2+^ and CTS could be assigned in other groups instead of the amino groups of CTS, because of unchanged results with the blocked amino group crossing the crosslinking. Moreover, comparing nCTS and GLA-cCTS, there is an increase in the maximum adsorption capacity despite the blocked amino group crossing the crosslinking (Table 2), the product containing imine bonds was created from the reaction of GLA and the amino groups of CTS, which is capable to adsorb Hg^2+^. As such, the adsorption mechanism does not belong only on the amino groups of CTS, but also other groups via the crosslinking. Moreover, the use of ECH or GLA in the crosslinking of CTS resulted in more affinity for Hg^2+^ than for Cu^2+^ [51]. On the other hand, the impact of crosslinking in CTS has also been examined in the removal of Cr^6+^ from aqueous solution, resulting in that the maximum adsorption capacity of GLA-cCTS was lower than that of both nCTS and ECH-cCTS at pH 6.0, and that the maximum adsorption capacity of ECH-cCTS was the highest (1420 mg g^−1^) [52] (Table 2). Hence, it shows that the Cr^6+^ adsorption process is assigned mainly on the amino groups of CTS.

**Table 2:** Published papers in heavy metal removal using AMs

AMs	Adsorbents	Heavy metal ions	Removal efficiency (%)	Maximum adsorption capacity (mg g^−1^)	pH	Recycling method	Desorption efficiency (%)	Recycle number	Refs.
PMs	CTS/PEG nCTS ECH-cCTS GLA-cCTS nCTS ECH-cCTS GLA-cCTS CTS PVT-*co*-PAN PVA/PEI CTS/PVA/PEI CA/PEI	Fe^2+^, Mn^2+^ Hg^2+^ Hg^2+^ Hg^2+^ Cr^6+^ Cr^6+^ Cr^6+^ Cu^2+^ Cu^2+^ Pb^2+^, Cd^2+^, Cu^2+^ Cd^2+^, Cu^2+^, Ni^2+^ Cu^2+^	– 15.1 18.3 35.2 37.9 45.5, 92.0 86.9, 31.7 97.2 87.4 60.0, 26.0, 16.0 74.8, 57.4, 50.3 14.8	38.0, 18.0 25.3 30.3 75.5 885.0 270.0, 1420.0 950.0, 347.0 87.5 44.3 122.2, 37.1, 29.9 112.1, 86.1, 75.5 7.4	5.0, 5.9 6.0 6.0 6.0 6.0 2.0, 6.0 2.0, 6.0 5.0 5.0 5.0 6.0 5.0	0.1 M HCl 1 M NaCl, 10^–4^ M EDTA 1 M NaCl, 10^–4^ M EDTA 1 M NaCl, 10^–4^ M EDTA 1 M NaCl 1 M NaCl 1 M NaCl 0.03 M H_2_SO_4_ 0.25 mM HCl, 0.25 mM EDTA 0.05 M HCl, 0.05 M HNO_3_ 0.05 M Na_2_EDTA 0.1 M HCl	> 98.0 73.1, 52.3 86.6, 50.5 43.2, 19.9 48.6 77.2, 49.1 40.3, 35.7 ~ 35.0 ~ 78.0, ~ 96.0 98.8, 61.8 (Cu^2+^) ~ 80.0 10.5	4 – – – 3 3 3 5 – 5 4 3	[48] [50] [50] [50] [52] [52] [52] [130] [129] [53] [54] [55]
PCMs	PANOB CMC Al oxide-CMC Fe oxide-CMC Fe oxide-PE_8_M Fe oxide-PE_25_M CPBC PANlB CCPM	Cu^2+^, Zn^2+^, Cd^2+^ Se^4+^ Se^4+^ Se^4+^ Cr^6+^ Cr^6+^ Cu^2+^, Zn^2+^, Cd^2+^, Ni^2+^ U^6+^ Cu^2+^, Pb^2+^	99.8, 98.9, 97.4 19.0 19.0 15.0 95.0 84.0 95.0, 95.0, 85.0, 70.0 70.5 96.0, 99.5	77.4, 65.4, 52.6 18.4 17.2 8.2 8.8 7.7 88.5, 72.9, 51.5, 48.5 14.1 9.6, 19.9	2.0–7.0 4.0–4.5 4.0–4.5 4.0–4.5 1.0–9.0 1.0–9.0 6.0, 7.0, 6.0, 8.0 5.0 6.5, 6.0	0.1 M HCl NaCl NaCl, NaOH – HCl, NaOH – 0.05 M Ca(NO_3_)_2_, 0.05 M EDTA, 0.05 M DTPA 0.1 M HCl 0.1 M HNO_3_; 0.01 M EDTA	> 90.0 11.0 15.0, 44.0 – 90.0 – > 90.0 ~ 68.0 72.1, 66.7; 75.3, 68.0	4 – – – – – – 7 –	[58] [59] [59] [59] [60] [60] [65] [66] [67]
ENMs	AOPAN/RC PEI/PVA PEI/PES TC PAN/PVA PVA CTS PVA/CTS CNC/CTS/PVA-SH PAN/CTS PEI/CTS	Fe^3+^, Cu^2+^, Cd^2+^ Cu^2+^, Cd^2+^, Pb^2+^ Pb^2+^, Cu^2+^, Cd^2+^ Cu^2+^, Cd^2+^, Pb^2+^ Cr^6+^, Cd^2+^ Cu^2+^, Pb^2+^ Ni^2+^, Cu^2+^ Ni^2+^, Co^2+^ Cu^2+^, Pb^2+^ Cd^2+^, Pb^2+^ Cr^6+^, Cu^2+^, Co^2+^	31.2, 11.5, 2.9 23.0, 25.6, 32.8 90.4, 89.8, 93.2 4.9, 4.6, 2.2 33.3, 28.0 – 46.0, 68.7 79.3, 77.1 39.4, 44.8 51.1, 52.9 50.33, 27.3, 19.3	417.2, 270.7, 127.0 70.9, 121.9, 94.3 94.3, 161.3, 357.1 49.0, 45.9, 22.0 66.5, 33.6 58.3, 161.7 10.3, 25.6 23.9, 10.0 484.1, 323.5 385.0, 240.0 139.0, 69.3, 68.3	2.0, 5.0, 6.0 5.0 5.0–7.0 2.0–7.0 2.0, 6.0 – 5.0 6.0 6.0 7.0, 5.0 2.0, 4.0, 6.0	0.1 M HCl 0.05 M EDTA, 0.05 M HCl, 0.05 M HNO_3_ 0.05 M EDTA – 0.1 M NaOH, 1 M HNO_3_ – EDTA – 4 M HCl – 0.01 M HCl, 0.01 M NaOH	> 70.0 95.6, 51.4, 20.7 (Cu^2+^) 96.2, 98.2, 97.2 – > 90.0 – 32.2, 41.8 – 90.6, 90.2 – 40.3, 21.0, 13.3	5 3 3 – 3 – 3 – 4 – 5	[84] [86] [89] [98] [85] [81] [96] [94] [97] [100] [99]
NEMs	CCGO GO/PSf GO/PVA GO/cellulose AgNPs-St-PEG-AcANCH PAN/MO/CTS PAN/MO pHEMA/CTS CTS/HAp CTS/PVA/Zeo MOFs Zr-MOFs	Cr^6+^ Cu^2+^, Cd^2+^, Pb^2+^, Cr^6+^ Cu^2+^, Ni^2+^, Cd^2+^ Co^2+^, Ni^2+^, Cu^2+^, Zn^2+^, Cd^2+^, Pb^2+^ Hg^2+^ Cd^2+^, Pb^2+^ Cd^2+^, Pb^2+^ Cd^2+^, Pb^2+^, Hg^2+^ Pb^2+^, Co^2+^, Ni^2+^ Cr^6+^, Fe^3+^, Ni^2+^ Cu^2+^ Cu^2+^	81.2 > 90.0 72.6, 62.3, 83.8 > 90.0 (from Co^2+^ to Cd^2+^), 100.0 (Pb^2+^) ~ 90.0, 85.0 61.2, 86.0 36.4, 77.2 37.6, 25.1, 78.4 78.5, 80.0, 55.5 ~ 100.0, 93.0, 98.0 6.0 98.8	67.7 75.0, 68.0, 79.0, 154.0 72.6, 62.3, 83.8 15.5, 14.3, 26.6, 16.7, 26.8, 107.9 158.2, 182.5 461.0, 390.0 91.0, 193.0 18.5, 22.7, 68.8 296.7, 213.8, 180.2 8.8, 6.2, 1.8 59.8 988.2	3.0 6.5, 6.4, 6.7, 3.5 5.7 4.5 7.0, 6.0 7.0, 5.0 7.0, 5.0 5.0 – – 6.0 6.0	0.1 M NaOH DI H_2_O/HCl 1 M HCl 0.1 M HNO_3_ – – 0.01 M HNO_3_ 1 M HNO_3_ DI H_2_O – –	76.9 ~ 90.0 64.7, 54.8, 75.2 ~ 98.0 (Pb^2+^) – – > 95.0 75.4, 72.5, 52.4 99.0, 92.0, 96.0 – –	5 3 6 10 – – – 5 5 5 – –	[118] [120] [26] [121] [128] [100] [100] [122] [123] [104] [124] [124]

*nCTS* natural CTS, *ECH-cCTS* ECH crosslinked CTS, *GLA-cCTS* GLA crosslinked CTS, *PVT* poly(vinyl tetrazole), *PANOB* PAN/organobentonite, *CMC* CTS/montmorillonite composite, *Al oxide-CMC* Al_2_O_3_/CTS/montmorillonite composite, *Fe oxide-CMC* Fe_3_O_4_/CTS/montmorillonite composite, *Fe oxide-PE*_*8*_*M* Fe_3_O_4_/PEI800/montmorillonite, *Fe oxide-PE*_*25*_*M* Fe_3_O_4_/PEI2500/montmorillonite, *CPBC* CTS-grafted-PAA-bentonite composites, *PANlB* poly(aniline) modified bentonite, *CCPM* crosslinked CTS/Al_13_-pillared montmorillonite, *AgNPs-St-PEG-AcANCH* AgNPs-base starch/PEG-PAA nanocomposite hydrogel, *MO* metal oxide, *DI H*_*2*_*O* distilled water, *HCl* hydrochloric acid, *HNO*_*3*_ nitric acid, *NaOH* sodium hydroxide, *NaCl* sodium chloride, *EDTA* ethylenediaminetetraacetic acid, *Na*_*2*_*EDTA* EDTA disodium salt, *H*_*2*_*SO*_*4*_ sulfuric acid, *Ca(NO*_*3*_*)*_*2*_ calcium nitrate, *DTPA* diethylenetriaminepentaacetic acid

Poly(ethyleneimine) (PEI) is considered a chelating agent and is useful for heavy metal removal from wastewater due to its high affinity to various metal ions. For example, a semi-interpenetrating polymer network of crosslinked poly(vinyl alcohol) (PVA)-matrix and PEI-complexing polymer was employed for the removal of Cu^2+^, Pb^2+^, and Cd^2+^ from aqueous solution [53], resulting in an affinity order for this membrane as Pb^2+^  > Cu^2+^  > Cd^2+^, which was similar with the range of maximum adsorption capacity of the ions per gram of membrane of 0.59, 0.47, and 0.33 m mol g^−1^, respectively. Supplementing PEI into a CTS/PVA mixture yields an increase of a large number of amine groups, which led to an increase in adsorption sites and increased its adsorption capacity [54]. The results indicated that the adsorbate removal efficiency of the CTS/PVA membrane with incorporated PEI (0.5 wt.%) was higher than the pristine membrane (40.0%). The adsorption capacity of CTS/PVA incorporated PEI-based PMs was 112.1, 86.1, and 75.5 mg g^−1^ for Cd^2+^, Cu^2+^, and Ni^2+^, respectively. For the heavy metal adsorption mechanism in these membranes, the large number of amino groups would be liable for the uptake of three heavy metal ions (-NH_3_^+^  + M^2+^ − NH_2_M^2+^  + H^+^; M^2+^ was Cd^2+^, Cu^2+^, and Ni^2+^) at a low pH value (pH < 7.75). However, supplementing too much PEI decreased membrane porosity and limited membrane efficiency. In other studies, PEI was mixed with cellulose acetate (CA), which was crosslinked by polyisocyanate [55] for the removal of Cu^2+^. It showed that the maximum adsorption capacity is 7.4 mg g^−1^ for Cu^2+^. It also showed that the BSA on CA/PEI-based PMs with and without chelating Cu^2+^ were 86.6 and 43.8 mg g^−1^, respectively. Overall, adsorption is considered a promising method for various pollutant removal from wastewater, owing to its simplicity, high removal efficiency, and low-cost.

### Applications of PCMs

**Fig. 6:**
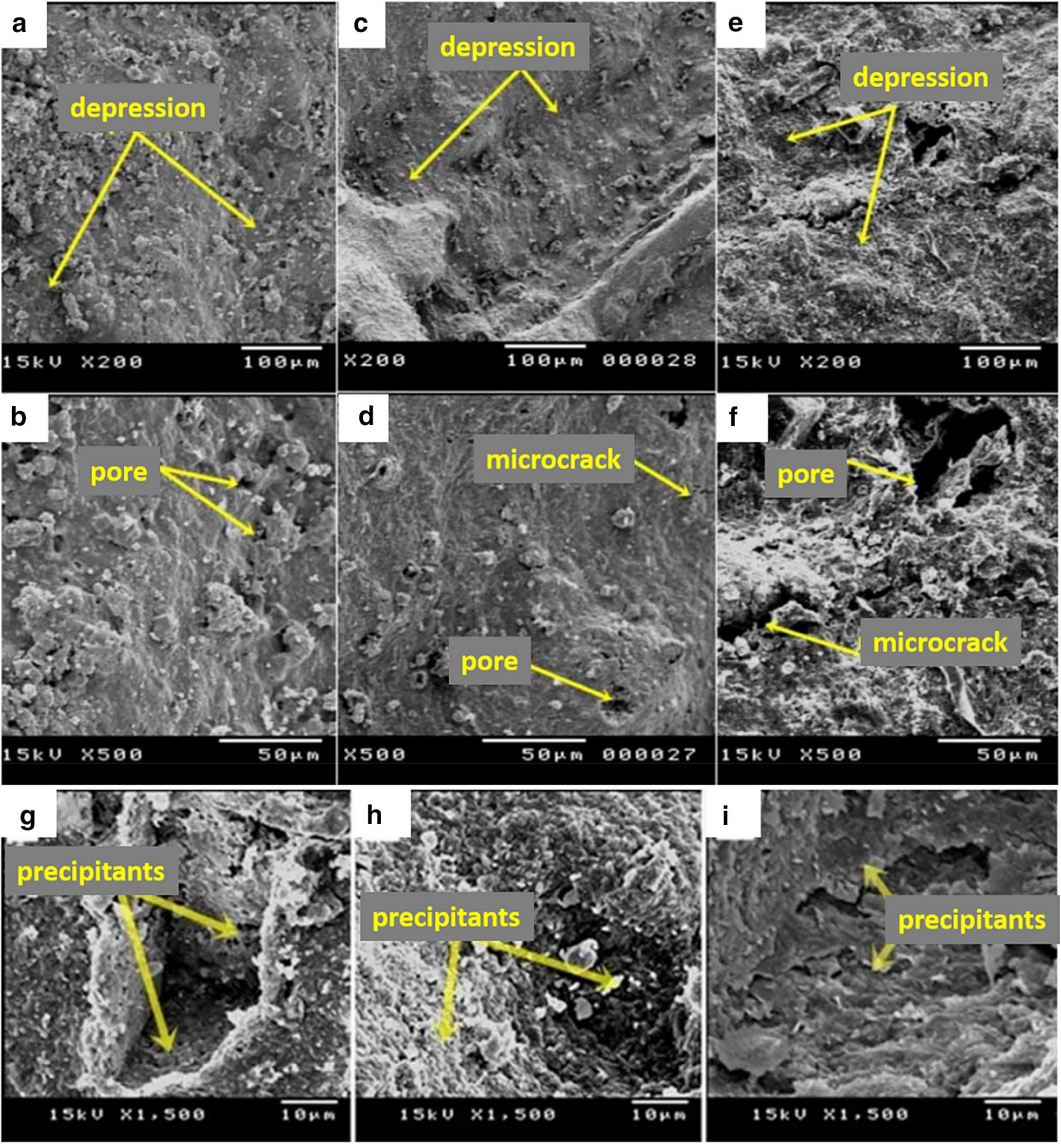
SEM images magnified to ×200 and ×500 from top-surface of PCMs before filtration; **a**, **b** 0.5% sawdust, **c**, **d** 2.0% sawdust, and **e**, **f** 5.0% sawdust, respectively. SEM images magnified to ×1500 from pores of PCMs after filtration; **g** 0.5% sawdust, **h** 2.0% sawdust, and **i** 5.0% sawdust. (Reprinted from [57])

**Fig. 7:**
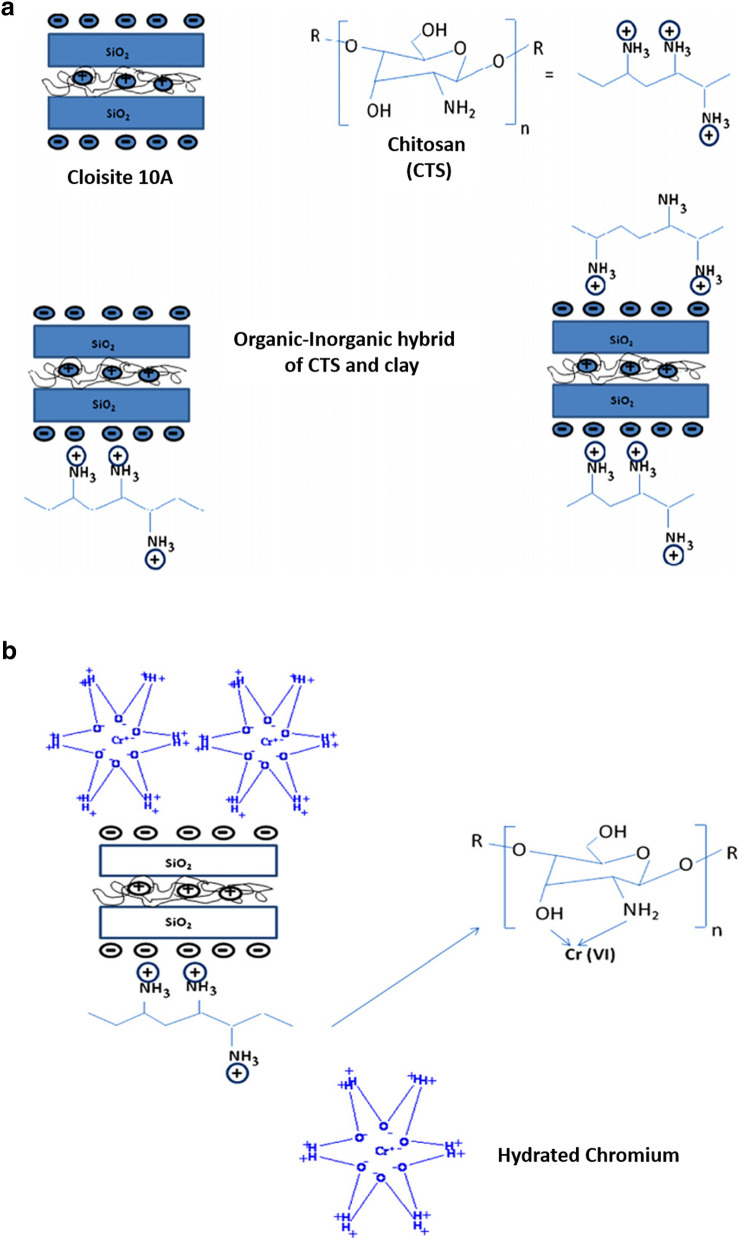
**a** CTS/clay-based Organic–inorganic hybrid, and **b** the interaction mechanism of CTS/organoclay and Cr^6+^. (Reprinted from [61])

In comparison to PMs, PCMs have some disadvantages, such as foul, slower, and more extreme recovery methods, while their thermal stability limits PMs. Nevertheless, natural clay materials (bentonite, kaolinite, and montmorillonite) are more useful for heavy metal removal owing to their lamellar structure, non-toxicity, low-cost, high cation exchangeability, and mechanical and chemical stabilities. Otherwise, their lamellar structures are stacked, which leads to unreachable sites and low surface areas. The natural clay materials combine with polymer materials to supplement the polymer materials or peel the clay sheets for unreachable scupper sites and to support good dispersion for crossing the drawback [56]. Good dispersion in the nanocomposite requires electrostatic interactions between the negatively charged surface of the layered clay materials and polymers. For instance, Ali et al. investigated the mixture of clay/sawdust to remove Pb^2+^, Cu^2+^, and Cd^2+^ from water [57]. The results suggest that removal efficiency was high and up to 99.0% for these heavy metals based on chemical analysis and scanning electron microscope (SEM) images (Fig. 6). Similarly, the amidoxime functionalized organobentonite/poly(acrylonitrile) (PAN) composite (PANOB) was synthesized and exerted to remove Zn^2+^, Cu^2+^, and Cd^2+^ [58], which resulted in maximum monolayer adsorption capacities based on the Langmuir model (77.4 mg g^−1^ for Cu^2+^, 65.4 mg g^−1^ for Zn^2+^, and 52.6 mg g^−1^ for Cd^2+^ at 30 °C), owing to the chelating PANOB with amidoxime (-C(NH_2_) = NOH) groups. The adsorption process also relates to the complexation of the surface and ion exchange progress [58]. In other research studies, incorporation of various polymers and montmorillonite-based composite materials, such as CTS–montmorillonite (CMC) [59] and PEI–montmorillonite (PEM) composites [60] have been used to remove Se^4+^ and Cr^6+^ from water at extra-low concentrations. The combination of metal oxide (Al oxide and Fe oxide) and these composite materials were recorded in Table 2. Specifically, a representative CMC and its interactions with Cr^6+^ are presented in Fig. 7 [61]. CTS-perlite composite was also proved for the efficient removal of Cu^2+^ (104.0 mg g^−1^) [62], Cd^2+^ (178.6 mg g^−1^) [63], and Ni^2+^ (114.9 mg g^−1^) [64]. Furthermore, a montmorillonite rich bentonite was graft-copolymerized with CTS [65]. It is shown that the immobilization of heavy metals in soils was achieved and was potentially a sustainable and cheap environmental technology. Polyaniline (PANI)/bentonite (PANlB) was fabricated by plasma polymerization of aniline onto the surface of bentonite [66], which was employed to remove U^6+^ from the aqueous solution. In another research study, crosslinked CTS/Al_13_-pillared montmorillonite (CCPM) was employed to conduct the removal of Cu^2+^ and Pb^2+^ of 9.6 and 19.9 mg g^−1^, respectively [67].

Also, depending on the aim of the studies, the combination of processes is considered as a further effective method comparing to an individual process. For instance, the combination of ion exchange and chemical precipitation treatments was used for the removal of heavy metal ions from contaminated water [68]. Moreover, this combination of the above processes was applied to remove Ni^2+^ ion with better efficiency (94.2–98.3%) than that of the individual ion exchange process (< 74.8%) [69]. Moreover, ceramic filters are effectively employed to remove heavy metals from aqueous solution owing to their higher flux, better durability, sharper pore size distribution, and higher damage tolerance than those of organic hollow fibers [70]. Generally, the adsorption capacity on PCMs is almost lower than that of PMs (Table 2), due to the significant functional groups in synthesized polymers (or biopolymers) [43–45].

### Applications of ENMs

**Fig. 8:**
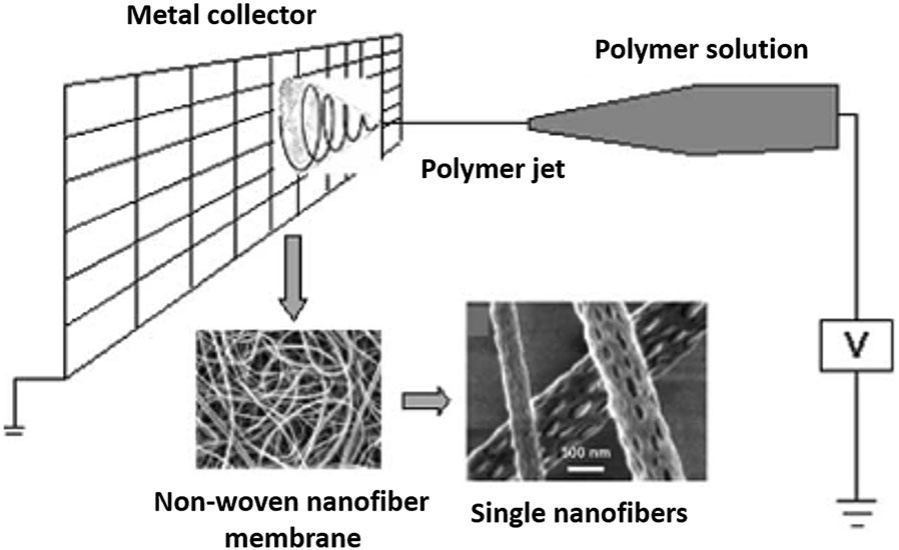
Scheme of basic electrospinning. (Reprinted from [78])

Electrospinning is a known method for creating fibers with nanometer to micron diameters into long polymeric fibers to obtain nanofiber membranes (nanostructure membranes) with a large ratio of surface to volume (10.0–40.0 m^2^ g^−1^), and high porosity (> 90.0%) [71, 72]. Generally, there are some advantages in electrospinning method, such as (i) wide selection in the material; (ii) high control for nanofiber diameter, microstructure, and arrangement; (iii) easy to incorporate additives in nanofibers; (iv) one-step and apparent process; (v) high porosity membranes (> 90.0%) and high surface-to-volume ratio; and (vi) practicability in creating nanostructures. Besides, there are also several disadvantages, such as (i) difficult to get nanofibers with diameters below 100 nm; (ii) difficult to possess maximum pore sizes smaller than 100 nm in ENMs; and (iii) slow yield speed. In particular, published patents of Formhals [73–77] describe the production of polymeric fibers using an electrostatic force. An electrospinning interpretation of polymeric nanofibers is shown in Fig. 8 [78]. Poly(acrylic acid) (PAA) is considered a significant polyelectrolyte, or metal ion complexing agent due to carboxyl groups on its chain [79]. Xiao et al. [80] examined the influence of electrospinning ultrafine PAA/PVA nanofibers and reported that the PAA/PVA nanofiber membrane was competent and fast in removing Cu^2+^ ion (91.0%) from aqueous. PVA is a known water-soluble polymer with a common chemical formula [CH_2_CH(OH)]_n_. it is required to maintain its hydrophilicity property, mechanical stability, and adsorption capacity to apply for the removal of heavy metal. Ullah et al. [81] used PVA with a crosslinking agent to remove heavy metals, and achieved adsorption capacity for Cu^2+^ and Pb^2+^ ions of 28.3 and 161.7 mg g^−1^, respectively.

Cellulose acetate (CA) is commonly used in nanofiltration membranes due to its comparatively high modulus, adequate flexural, and tensile strength [82]. CA is modified with carboxylate groups to assist in binding heavy metal ions by complexation of surface mechanisms. For instance, Tian et al. prepared poly(methacrylic acid) (PMAA)/CA nanofiber membranes to remove Hg^2+^, Cu^2+^ and Cd^2+^ ions [83]. The results suggest that its adsorption capacity extends with increasing initial pH value. Moreover, its adsorption selectivity for Hg^2+^ was quite high. Saturated ethylenediaminetetraacetic acid (EDTA) solution could also be applied for heavy metal removal from the membrane surface and recycling performance. Feng et al. fabricated PAN/CA nanofiber membranes and amidoxime polyacrylonitrile/regenerate cellulose (AOPAN/RC) nanofiber membranes to remove Fe^3+^ (417.2 mg g^−1^), Cu^2+^ (270.7 mg g^−1^), and Cd^2+^ (127.0 mg g^−1^) ions from wastewater [84]. The results showed that their desorption rate of Fe^3+^, Cu^2+^, and Cd^2+^ ions conserved more than 80% of the first desorption rate after undergoing five adsorption and desorption cycles, and excellent reusability of AOPAN/RC composite nanofiber membranes. However, the adsorption capacity of Cr^6+^ and Cd^2+^ ions achieved 66.5 and 33.6 mg g^−1^ on PAN/PVA membrane [85]. It indicated that this result in the removal of Cd^2+^ ion was lower than that of the AOPAN/RC membrane.

**Fig. 9:**
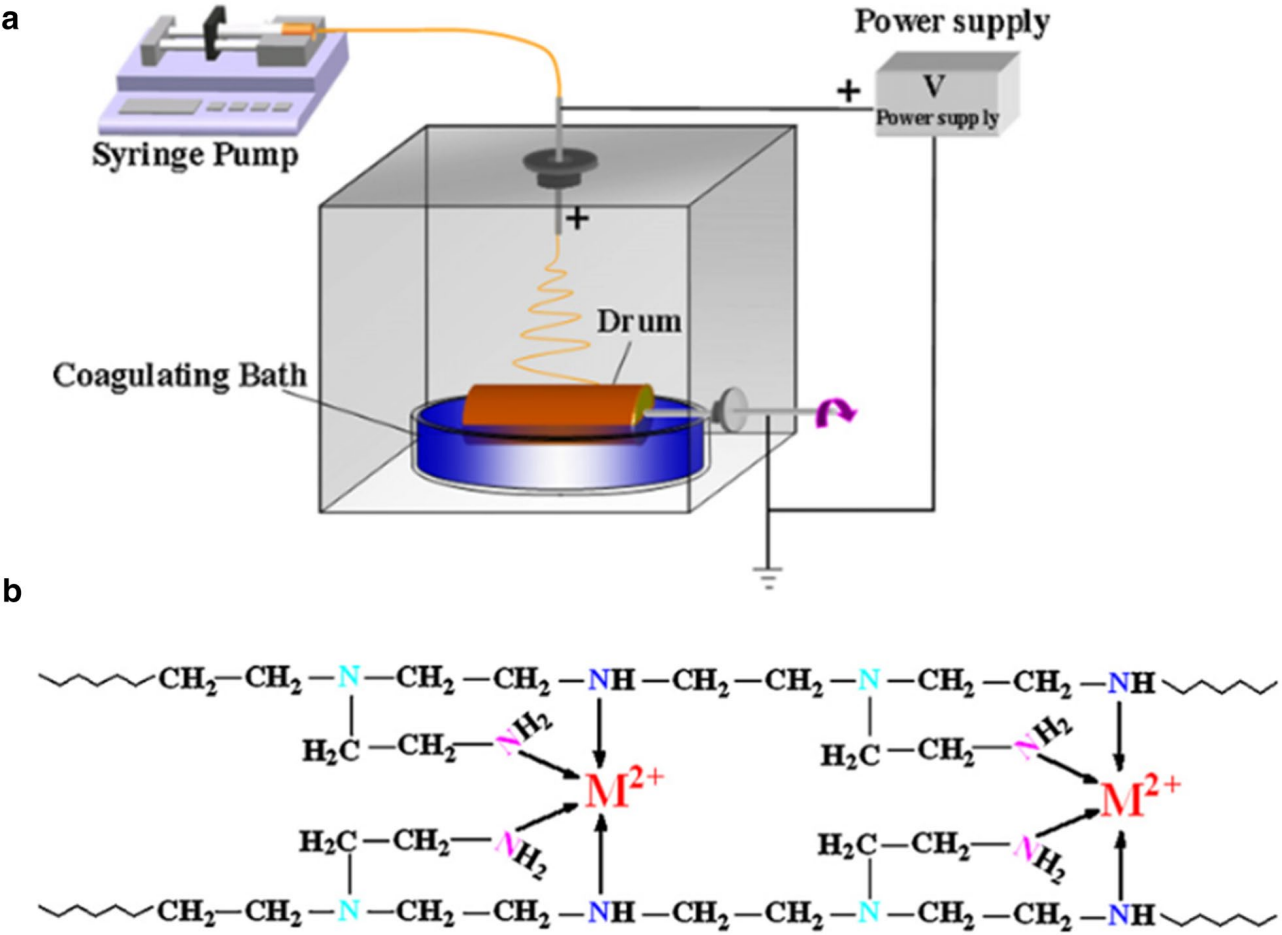
**a** Scheme of a wet-electrospinning process and **b** removal mechanism of heavy metal ions (M^2+^) using N atoms in PEI polymer chains. (Reprinted from [86])

PEI/PVA nanofibers affinity membranes were prepared by a special wet-electrospinning process [86] (Fig. 9a). As known, for the normal electrospinning process, ENMs with designed thicknesses are created via a spinneret electrospinning system. The wet-electrospinning process is complex more than the normal electrospinning one, it means that the nanofibers crystallization in ENMs is generated by removing the residual solvent in a coagulation bath. Generally, wet-electrospinning was done in a liquid medium in which the desired solvent is available to maintain and control the surface affinity. For example, based on the metal ion’s character, surface modification, such as surface affinity through the functional groups or by additional modifier is required. In a wet-electrospinning process, surface modification is done easily because a modifier can be added at the liquid medium (coagulation bath). Furthermore, crystallization can be controlled to the desired level by removing the residual solvent. It means surface properties can be tuned for increasing the adsorption properties of heavy metals ion. For the case of the normal electrospinning method, controlling surface modification and crystallization are limited compared to the wet-electrospinning process. In this case, the only chance of surface modification is mixing modifiers in the spinneret through the bypass or coaxial system. But in the wet-electrospinning, three options, such as spinneret, liquid medium (coagulation bath), and crystallization by residual solvent removal. Many of the cases, mixing modifiers in the spinneret for normal spinning process make trouble for the spinning because it changes the solution properties, which make a barrier for the spinning process as well as surface properties of the nanofiber, which is not expected. Furthermore, this wet-electrospinning method would probably reduce both costs and times in the manufacturing process with used crosslink agents. The resultant membrane (obtained nanofibers affinity membranes) strong adsorption capacity for Cd^2+^, Pb^2+^, and Cu^2+^ ions and maximum adsorption capacity from isotherm of 121.95, 94.34, and 70.92 mg g^−1^, respectively [86]. Additionally, these resultant adsorption capacities are also related to an affinity order of these heavy metal ions for this membrane as above-mentioned of PVA/PEI membrane-based PMs. This adsorption mechanism is based on the interaction between the nitrogen (N) atoms in the PEI chains and the three heavy metal ions (Fig. 9b). Specifically, the nitrogen atom has a lone pair of electrons that bind a metal ion via the sharing of an electron pair to create a metal complex [87, 88]. Besides, it was reused in the EDTA solution after the first adsorption process. Therefore, its adsorption capacity could also be recovered. Furthermore, PEI was also incorporated with poly(ether sulfones) (PES) to form a nanofiber membrane [89] for the pseudo-second-order model. Its intra-particle diffusion process was the rate-limiting step of the adsorption process. The results suggest that the maximum adsorption capacity values were 94.34, 161.29, and 357.14 mg g^−1^ for Pb^2+^, Cu^2+^, and Cd^2+^ ions, respectively (pH = 5–7). Besides, these resultant adsorption capacities are also related to an affinity order of these heavy metal ions for this membrane as above-mentioned of PVA/PEI membrane-based PMs. The adsorption equilibrium all conformed to the Langmuir isotherm equation. The adsorption mechanism of PEI/PES nanofiber membrane was focused on the amine groups of PEI chains at higher pH values (pH = 5–7, lower concentration of proton). The electrostatic repulsion of heavy metal ions was reduced due to lesser competition between the heavy metal ions and protons for the amine groups. The heavy metal ions could constitute together to form bonds with the unpaired electrons of the amine groups.

**Fig. 10:**
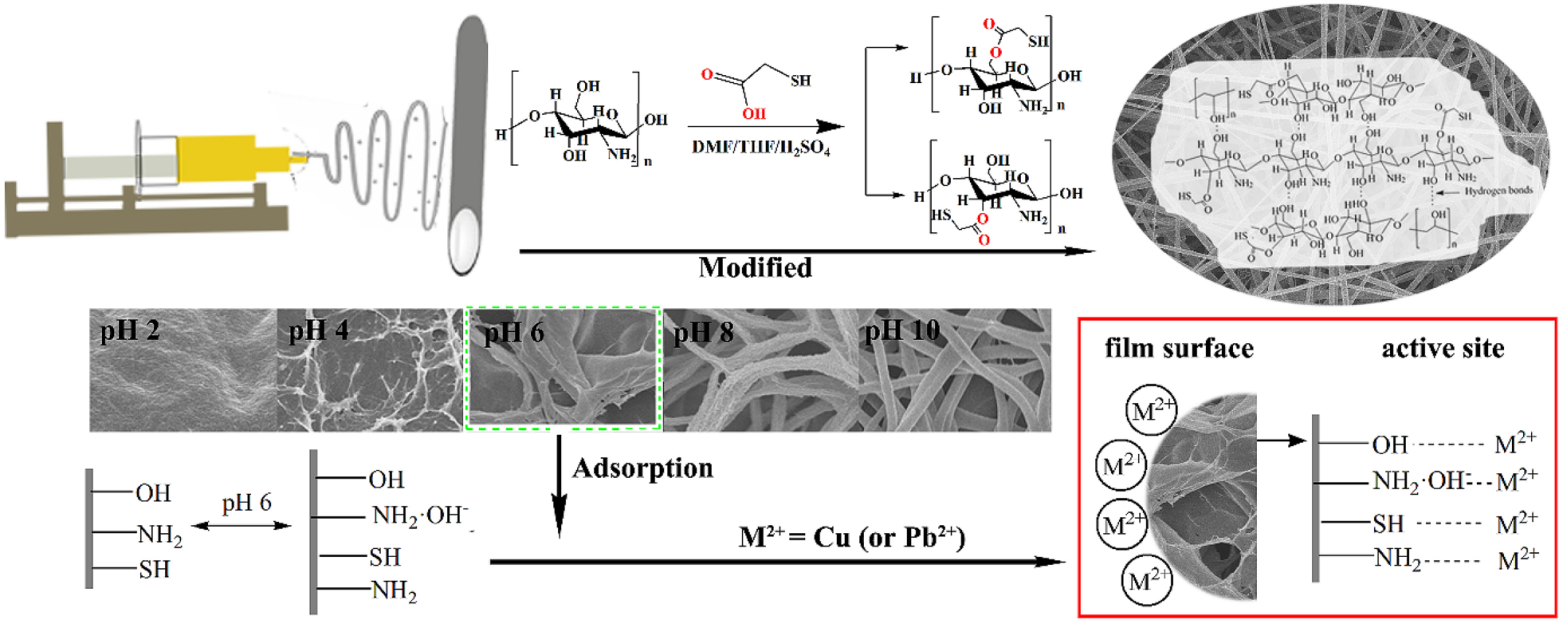
Electrospinning CNC/CTS/PVA-SH composite nanofiber membrane. (Reprinted from [97])

CTS is considered as a highly stable molecule and difficult to degrade due to amino groups along the CTS chain, which could be used for heavy metal removal from wastewater [90]. CTS could remove heavy metal ions by two adsorption mechanisms. The first mechanism is chelation and formation of the CTS–ion complex via intra- and inter-CTS chains. The second mechanism is electrostatic interaction, as CTS could become positively charged in an acid condition. However, there are some disadvantages in the electrospinning method of pure CTS, which include high hydrogen bonding in intra- and inter-CTS and poor solubility in most organic solvents. Several scientists combined poly(ethylene oxide) (PEO) with CTS to enhance the spin capability of CTS [91–93]. For instance, Aliabadi et al. [92] showed that the adsorption selectivity on the membrane was Pb^2+^  < Cd^2+^  < Cu^2+^  < Ni^2+^ and its reusability for various metal ion removal was investigated after five adsorption–desorption cycles. CTS/PEO/permutit (PT) composite nanofiber membrane was a potential absorbent in Cr^6+^ ion removal from aqueous solution [93], which resulted in nanofibers that were homogeneous and bead-free, and had strong interactions among CTS, PEO, and PT. All the results indicated that the electrospinning CTS/PEO/PT nanofibers could be used as the promising absorbents for the removal of Cr^6+^ ion. In other research studies, PVA/CTS nanofiber membrane adsorption kinetics was inspected for the removal of Ni^2+^ and Co^2+^ ions [94]. The results suggest that it fitted the adsorption data to the pseudo-first-order and that the adsorption capacities of Ni^2+^ and Co^2+^ ions were 23.9 and 10.0 mg g^−1^, respectively. On the contrary, Cheng et al. [95] presented the pseudo-second-order model for the removal of Cu^2+^ ion on a modified CTS membrane by a chemical-controlling adsorption mechanism. The adsorption of Cu^2+^ on this membrane consisted of chelation ion-exchange progress due to the presence of –NH_2_ groups in CTS chains. Specifically, at pH values of 5–6, it was a condition that caused the disadvantage for electrostatic interaction between Cu^2+^ ion and this membrane, due to its positive surface charge. Thus, this mechanism was used for adsorption of Cu^2+^ ion onto the membrane via a complex of inner-sphere, which was surface chelating ion exchange instead of electrostatic interaction. A setting bonding of Cu^2+^ and Ni^2+^ ions on the macroporous CTS membrane was also discovered by Ghaee et al. [96] by the pseudo-second-order model. The results suggest that the adsorption of Cu^2+^ ion (25.6 mg g^−1^) was higher than that of Ni^2+^ ion (10.3 mg g^−1^) with the same initial concentration condition, which indicated that the affinity of this membrane for binding Cu^2+^ ion was better than for binding Ni^2+^. The comparison between the PVA/CTS membrane [94] and the CTS membrane [96] shows that the adsorption capacity of Ni^2+^ ion on PVA/CTS was higher than that of CTS membrane owing to the incorporation of PVA. Furthermore, Wang et al. [97] prepared cellulose nanocrystals (CNC)/CTS/PVA nanofiber membranes with thiol-functionalized (CNC/CTS/PVA-SH) (Fig. 10). The results showed that the removal of Cu^2+^ (484.1 mg g^−1^) and Pb^2+^ (323.5 mg g^−1^) ions was best with 5.0 wt% of CNC (pH = 6, t = 4 h). The removal was conducted primarily based on the chemical adsorption mechanism. This result in the removal of Cu^2+^ ion was higher than the CTS membrane (25.6 mg g^−1^) [96], the thiol-functionalized cellulose (TC) membrane (49.0 mg g^−1^) [98], and the PEI/CTS membrane (69.3 mg g^−1^) [99]. The removal of Pb^2+^ ion was also better than both the TC membrane (22.0 mg g^−1^) [98] and the PAN/CTS membrane (240.0 mg g^−1^) [100] (Table 2). Adding of CNC into electrospinning of polymer matrices (e.g.: CTS/PVA blend) improves the thermal and mechanical properties of nanofibers [101]. In contrast to their reactive sites were almost finite [102]. Adding amino, carboxyl, and thiol groups [83, 103] onto the electrospinning fiber surfaces improves the use of them in outstanding adsorption removal for heavy metal [104].

### Applications of NEMs

**Fig. 11:**
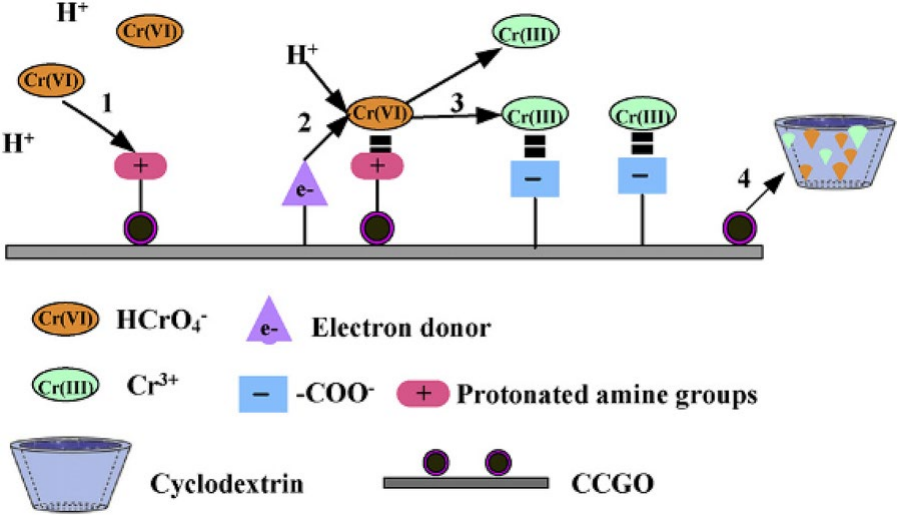
Removal mechanism of Cr^6+^ using cyclodextrin–CTS/GO (CCGO). (Reprinted from [118])

The unique structure and surface characteristics of nanomaterials (carbonaceous materials, nanometal or nanometal oxides, and other organics) enable use as adsorbents for heavy metal removal [105–112] (e.g.: larger surface contact, higher reactivity, and better disposal ability). Carbonaceous materials (carbon nanotubes (CNTs), active carbon (AC), and graphene) are known as potential counterparts in polymer-based composites owing to their high aspect ratio, mechanical strength, compatibility of the carbon matrix with the polymeric structure, and strong interactions and adhesion [113–115]. Polysulfone (PSf)/AC composite membrane was fabricated by Said et al. [116] and showed that the concentration of AC plays a grave role in support of enhancing heavy metal removal from water. Compared with the above carbonaceous materials, owing to the presence of numerous functional surface groups, graphene oxide (GO) was more widely employed in polymer matrices to enhance heavy metal removal with both high surface area and water solubility [113]. For instance, GO was incorporated with CTS to produce hydrogel composites [117–119]. Li et al. attributed some important features of magnetic cyclodextrin–CTS/GO (CCGO) composite [118] (e.g.: numerous hydroxyl and amino groups, high surface area, and magnetic properties) to the discovery of the removal efficiency of Cr^6+^ ion (67.7 mg g^−1^) at low pH and the removal mechanism of Cr^6+^ ion on CCGO as shown in Fig. 11. Moreover, GO could also incorporate with other polymers (e.g.: PVA, PSf, and cellulose) [26, 120, 121] to employ the removal of many heavy metals. GO/PVA membrane [26] was used to conduct the removal of Cu^2+^, Ni^2+^, and Cd^2+^ with 72.6, 62.3, and 83.8 mg g^−1^ of the adsorption capacity, respectively. GO/PSf membrane [120] was used to remove Cu^2+^, Cd^2+^, Pb^2+^, and Cr^6+^ ions, suggesting that the adsorption capacity of them were 75.0, 68.0, 79.0, and 154.0 mg g^−1^. The oxygen-containing groups of this membrane and its water permeability and hydrophilicity could make it easy to adsorb metal ions and contribute to their interstitial diffusion. GO/cellulose membrane [121] was used for adsorption removal of Co^2+^, Ni^2+^, Cu^2+^, Zn^2+^, Cd^2+^, and Pb^2+^ ions with results of 15.5, 14.3, 26.6, 16.7, 26.8, and 107.9 mg g^−1^ of adsorption capacity. It indicated that their affinities for these metal ions had an order of Pb > Cu > Cd > Zn ≥ Ni ≥ Co. This affinity arrangement was suitable with the first stability constant of the associated metal hydroxide and acetate.

**Fig. 12:**
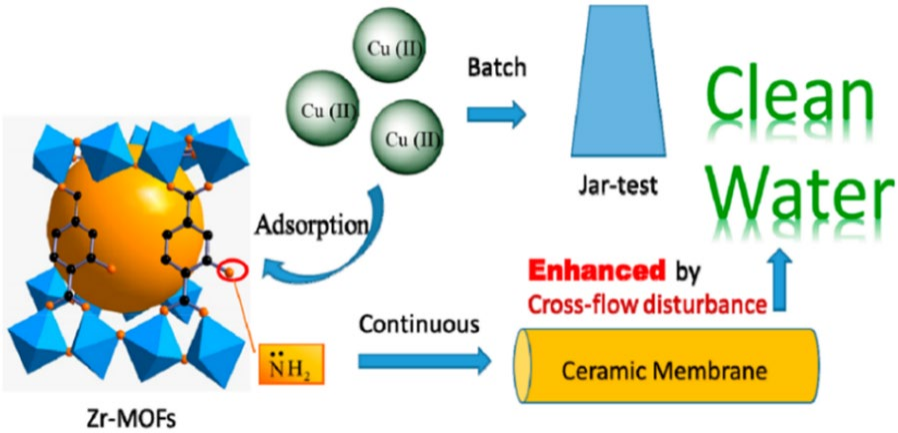
Cu^2+^ adsorption process onto Zr-MOFs via CM novel cross-flow disturbance. (Reprinted from [124])

In other research studies, CTS-based composite membrane separation of Cd^2+^, Pb^2+^, and Hg^2+^ from aqueous were carried out on poly(hydroxyethylmethacrylate) (pHEMA)/CTS composite membrane [122]. This suggests that maximum absorption capacity for Cd^2+^, Pb^2+^, and Hg^2+^ were 18.5, 22.7, and 68.8 mg g^−1^ within the equilibrium time of approximately 45 min. Also, Aliabadi et al. [123] investigated the removal efficiency of CTS/hydroxyapatite (HAp) composite nanofiber membranes for Pb^2+^, Co^2+^, and Ni^2+^ ions from water. The results suggested that their maximum adsorption capacities were 296.7, 213.8, and 180.2 mg g^−1^ within the equilibrium time near 30 min. CTS/PVA/zeolite (Zeo) nanofibers composite membrane was fabricated by Habiba et al. [104]. Their adsorption abilities were investigated for Cr^6+^, Fe^3+^, and Ni^2+^ ions, which were 8.8, 6.2, and 1.8 mg g^−1^, respectively [104]. The increase in ionic radius (Cr^6+^  < Fe^3+^  < Ni^2+^) led to decreasing adsorption capacity (Cr^6+^  < Fe^3+^  < Ni^2+^) at high concentration. This meant that the decreasing charge density with increasing ionic radius decreased the interactions of the active sites with this membrane [92], as well as decreasing the adsorption capacities of different metal ions. In another research, Wang et al. applied a novel cross-flow disturbance of PCMs to enhance the removal of Cu^2+^ ion on Zr-based metal − organic frameworks (Zr-MOFs) [124]. It indicated that Zr-MOFs removed Cu^2+^ (988.2 mg g^−1^, pH = 6, T = 40 °C), and its adsorption capacity was also higher than that of MOFs (59.8 mg g^−1^) at the same condition. The major benefits of this method are the enhancement of adsorption of Cu^2+^ ion on Zr-MOFs and the convenience in wastewater treatment (Fig. 12), which led to achieving an effective method for heavy metal removal from wastewater. The mechanism of adsorption removal of Cu^2+^ ion based on the chemical interactions between N atoms of this membrane (amidogen nitrogen atoms) and Cu^2+^ ion. It was explained that the lone electron pair of N was donated as a Lewis base to Cu^2+^ ion to create a complex coordinate covalent bond. Nevertheless, the hybrid membranes also displayed superb adsorption capacities for heavy metal ions in aqueous solution [125–128].

## Recycling performance of AMs

To evaluate the quality of the AMs for practical use, the avail oneself of these AMs have to be stable in chemical factor and reusable. Therefore, to maintain the adsorption capacity during the repeated use of the adsorbents in wastewater treatment or purification, recycling performance is seen as one of the important features as well as one of the advantages in the adsorption process. To identify their recycling performance in heavy metal ions removal, the desorbing solution method is one of the most common and simplest methods for the adsorbent regeneration; however, the selection of the desorbing agents in whole recycling process depends on the aim of the studies that still well maintain the adsorption capacity. The common desorbing agents are employed in this recycling method including sodium hydroxide (NaOH), sodium chloride (NaCl), calcium nitrate (Ca(NO_3_)_2_), hydrochloric acid (HCl), sulfuric acid (H_2_SO_4_), nitric acid (HNO_3_), ethylenediaminetetraacetic acid (EDTA), EDTA disodium salt (Na_2_EDTA), and diethylenetriaminepentaacetic acid (DTPA) aqueous solutions with different concentrations. There were lots of successful investigations in the recycling performance of AMs with a high desorption rate as well (Table 2). As above-mentioned, the EDTA, Na_2_EDTA, and DTPA aqueous solutions are considered as strong complexing agents for desorption of metal ions and achieved good-results in regeneration; however, their cost is seen as a disadvantaged to apply for the recycling process. To possess the economic factor for the recycling process, the inorganic acid/base (HCl, HNO_3_, and NaOH) and NaCl aqueous solutions were the most recommended owing to their inexpensive cost and desorption efficiency. A summary of the recycling performance of PMs is listed in Table 2.

Generally, the metal ion desorption mechanism in the HCl solution occurred mainly due to the ion exchange. For CTS/PEG membrane-based PMs [48], the adsorbed Fe^2+^ and Mn^2+^ ions on this membrane could be effectively desorbed (> 98.0%) in 0.1 M HCl solution for 6 h, as well as it could be repeated use in four cycles without loss of the adsorption capacity for these ions. However, in reality, both adsorption and desorption performances were decreased in the increasing of recycling steps. For example, the use of 0.1 M HCl resulted in about 30.0% reduction of the first cycle comparing to the second cycle in the Cu^2+^ ion desorption efficiency of the CA/PEI membrane-based PMs [55], but the difference between second and third cycles was kept at a low level. For ENMs, the use of 0.1 M HCl solution in Fe^3+^, Cu^2+^ and Cd^2+^ ions desorption led to resulting the desorption efficiencies of AOPAN/RC membrane [84] were 76.2%, 91.7%, and 90.3% after three cycles, respectively; in particular, these Fe^3+^, Cu^2+^ and Cd^2+^ ions desorption efficiencies still obtained higher than 70.0% after five cycles. It indicated that AOPAN/RC membrane-based ENMs obtained reasonably good reusability. Besides, after four cycles, CNC/CTS/PVA-SH membrane-based ENMs [97] the Cu^2+^ and Pb^2+^ ions desorption efficiencies were 90.6% and 90.2% in 4 M HCl solution, respectively, which could relate to the damage of the nanofibers membrane, as well as the presentation of residual heavy metal ions in the nanofibers membrane led to reducing the adsorption capacity during the desorption process. Due to intermolecular and intramolecular hydrogen bondings in the incorporation of CNC into the CTS/PVA, it led to the membrane more tightly bounded. Thus, the nanofibers membrane recyclability would be decreased with the increase of the recycling number. This reduction was attributed to the dissolved CTS compound of the nanofibers membrane under the concentrated acidic condition in the desorption process. Besides, PVA and CTS all were insoluble in water at room temperature, a stable hydrogen bonding structure could be created via these PVA and CTS molecules, which became further stable with the incorporation of CNC. Special for PANOB membrane-based PCMs [58], the heavy metal ion adsorption mechanism is not only ion exchange but also complexation by the interaction between amidoxime groups on this membrane and heavy metal ions. In the regeneration process, 0.1 M HCl solution was employed to reuse the spent adsorbent, it indicated that its desorption efficiency could obtain high values (> 90%) after four cycles. Moreover, the recycling performance of PANlB membrane-based PCMs is one of the important factors for its real application, Liu et al. [66] investigated its reusability in U^6+^ ion desorption process through 0.1 M HCl solution, which showed that PANlB achieved excellent stability after seven cycles. For NEMs, GO/PSf [120] and GO/PVA membranes [26] were regenerated in heavy metal ion solution through HCl solution. The results suggested that the recycling number of GO/PSf and GO/PVA membranes were three and six cycles, respectively. Special for GO/PVA membrane, after the sixth cycle, the adsorption capability for Cu^2+^, Cd^2+^ and Ni^2+^ ions reduced to approximately 10.0%, 12.0%, and 21.0%, respectively, which was probably involved to loss of binding sites after each desorption step.

As known, the metal complexes of PEI will be dissociated in acidic solution, then the protons will compete with the metal ions for donating N atoms. Therefore, to investigate this loss of desorption capacity and destruction of the membrane involved to the acidic treatment or not, Bessbousse et al. [53] conducted a comparison of HCl and HNO_3_ solutions in Cu^2+^ ion desorption efficiency of PVA/PEI membrane-based PMs in five cycles. The results indicated that the use of 0.05 M HCl is better than that of 0.05 M HNO_3_ (Table 2), due to degradative oxidation of the membrane for the desorption with 0.05 M HNO_3_. Besides, this is also similar to the results of Wang et al. [86] Cu^2+^ ion desorption efficiency of PEI/PVA membrane-based ENMs through the use of HCl and HNO_3_ solutions. However, since the nature of HNO_3_ is an oxidizing reagent and the nanofibers membrane could be degraded, Wang et al. selected EDTA (C_o_ = 0.05 M) is a strong complexing agent for Cu^2+^ ion desorption [86]. It is shown that the Cu^2+^ ion adsorption capability of the nanofibers membrane was recovered after regeneration. Additionally, the Cu^2+^ ion desorption efficiencies in 0.05 M EDTA were better than that of both 0.05 M HCl and 0.05 M HNO_3_ after three cycles, mainly due to the different mechanisms of the desorbing agents. Specifically, the desorption process in EDTA could create a stable complex with metal ions without much affecting its adsorption efficiency, while that one in HCl and HNO_3_ solutions occurred mainly the ion exchange. This is also agreed on in Cu^2+^ and Pb^2+^ ions desorption efficiency of CCPM membrane-based PCMs [67] in a comparing between 0.1 M HNO_3_ and 0.01 M EDTA aqueous solutions. It is similar to PVT-co-PAN membrane-based PMs as well [129], EDTA, and HCl (0.25 mM) solutions all were used for Cu^2+^ ion desorption. It showed that the Cu^2+^ ion desorption efficiency in 0.25 mM EDTA solution (~ 96.0%) was more effective than that in HCl solution (~ 78.0%). In other studies, the use of HNO_3_ in the metal ion desorption process obtained high desorption efficiency for NEMs. For instance, pHEMA/CTS membrane [122] obtained high desorption efficiency (> 95%) for Cd^2+^, Pb^2+^ and Hg^2+^ desorption process after five cycles (0.01 M HNO_3_). It means that the chelated metal ion spheres were broken led to releasing metal ions from the surface of solid material into the desorption medium. Besides, CTS/HAp membrane-based NEMs [123] also achieved five cycles in the recycling process without almost a significant loss in removal efficiency (1 M HNO_3_). Special for GO/cellulose membrane [121], the recycling number could be achieved to ten cycles and effective Pb^2+^ ion desorption performance (~ 98.0%) with the use of 0.1 M HNO_3_ desorbing agent.

Additionally, the use of EDTA solution in heavy metal ions desorption was almost effective, for instance, heavy metal ions-loaded PEI/PES membrane-based ENMs [89] could be regenerated successfully in 0.05 M EDTA solution, and its adsorption efficiency was not affected much. The results indicated that the Pb^2+^, Cu^2+^, and Cd^2+^ ions desorption efficiency achieved 96.2%, 98.2%, and 97.2%, respectively after the three cycles because amino groups on this membrane were considered one of the most effective functional groups to be applied for affinity application. In another study, EDTA was also used to desorb Cu^2+^ and Ni^2+^ ions from spent CTS membrane-ENMs [96] indicated that the Cu^2+^ and Ni^2+^ ions desorption percentage were 68.7% and 46.0% in the first cycle, respectively. Thereafter, these values reduced for the second and third cycles, probably due to the reduction of the driving force. Special for CTS/PVA/PEI membrane-based PMs [54], use of 0.05 M Na_2_EDTA aqueous solution could regenerate successfully for this membrane led to non-decreasing much in its adsorption efficiency (i.e.: only lesser 5% after four cycles), suggesting its potential values for both stability and reusability of the adsorption for Cd^2+^, Cu^2+^, Ni^2+^ ions.

Furthermore, EDTA (10^–4^ M) and NaCl (1 M) aqueous solutions were also considered to compare in Hg^2+^ desorption performance from nCTS, ECH-cCTS, and GLA-cCTS membrane-based PMs [50], It suggested that the use of NaCl aqueous solution was effective more than that of EDTA solution basing on the Hg^2+^ desorption performances because the stable complexes (HgCl_2_) and negative charge complexes (i.e.: HgCl_4_^2−^ and HgCl_3_^−^) were formed, while the formation of complexes with EDTA [i.e.: Hg(EDTA)^2−^] taken only a Hg^2+^ small fraction. In addition to above stable complexes formations, NaCl solution was used to desorb Hg^2+^ ion from these membranes also explained to be involved to the electrostatic attraction between the metal species and charged species from elution led to weakening this interaction between CTS and Hg^2+^ ion via the electric double layer compression contributing to promoting the Hg^2+^ ion desorption. Special for the GLA-cCTS membrane, it showed lower recovery efficiency comparing to other CTS membranes; however, this membrane was chemically stable further at lower pH values.

For the Cr^6+^ ion desorbing agent, the salt aqueous solution (NaCl) or combination of both acidic and basic aqueous solution (HCl or HNO_3_, and NaOH) was almost commonly employed for Cr^6+^ desorption process from spent AMs. For instance, nCTS, ECH-cCTS, and GLA-cCTS membranes-based PMs [52], 1 M NaCl aqueous solution was selected to desorb Cr^6+^ ion from these PMs. As above-mentioned, the metal ions adsorption on CTS could relate to different mechanisms including chelation, electrostatic attraction, ion exchange, etc., which depend on the pH values, the composition of the solution, and metal ions features. Baroni et al. indicated that the electrostatic attraction of Cr^6+^ ion with CTS was affected by the polymerization – deacetylation degree as well as the dispensation of acetyl groups along CTS chain. Therefore, NaCl solution was used to desorb Cr^6+^ ion from these membranes related to the electrostatic attraction between the metal species and charged species from elution led to weakening this interaction between CTS and Cr^6+^ ion via the electric double layer compression. The results suggested that the adsorption capacity of GLA-cCTS membrane decreased a little bit after the first cycle, and the desorption capacity of nCTS membrane decreased slightly in the third cycle. Besides, Larraza et al. selected Fe_3_O_4_–PEI800–montmorillonite (Fe oxide-PE_8_M) hybrid material-based PCMs [60] to investigate its regeneration for the Cr^6+^ desorption in basic aqueous solution (pH = 14), suggesting that desorption efficiency was about 90.0%. In another work, Liu et al. conducted regeneration for PAN/PVA membrane-based ENMs [85] by acidic and basic aqueous solutions (0.1 M NaOH and 1 M HNO_3_), Cr^6+^ and Cd^2+^ ions desorption performance of this nanofibers membrane could be regenerated and reused for three cycles, which was expressed by its regeneration efficiency of above 90.0% after three recycles. Liu et al. also showed that the nanofibers membrane was strongly durable owing to the completed nanofibers structure. Additionally, Yang et al. used 0.01 M HCl and 0.01 M NaOH solutions to regenerate the Cr^6+^, Cu^2+^ and Co^2+^ ions desorption from spent PEI/CTS membrane-based ENMs [99], resulting in that the desorption efficiency reduced 17.8%, 11.3% and 13.5% after five cycles respectively; moreover, their adsorption capacities were still highly maintained after five cycles. It could be explained through the covalent bondings between PEI and metal ions as well as hard to separate all from the sites of the adsorption during regeneration.

Furthermore, for CCGO membrane-based NEMs [118], the Cr^6+^ desorption efficiency was 76.9% in 0.1 M NaOH solution after five cycles, no visible change was observed. Besides, the Cr^6+^ adsorption capacity of CCGO was easy and fast owing to the magnetic property, resulting in that the decrease in the Cr^6+^ adsorption amount was less than 5.0% after five cycles. Li et al. also explained the slight decrease in Cr^6+^ removal efficiency during five cycles including: (1) due to cavities of cyclodextrin, Cr^6+^-loaded CCGO could not be desorbed effectively led to reducing its adsorption capacity with the rise of adsorbed Cr^6+^ remaining on those cavities; (2) Cr^6+^ was partly decreased into Cr^3+^ owing to the surface hydroxyl groups on CCGO; moreover, several Cr^3+^ was precipitated on the CCGO surface at low pH (i.e.: Cr_2_O_3_) led to decreasing the active sites on CCGO with the rise of Cr_2_O_3_ remaining on CCGO, as well as lesser adsorbed Cr^6+^ with the employed CCGO. Thus, the electrostatic interaction of CCGO with Cr^6+^ was poor involving to decrease in several -NH_2_ and -OH groups of CCGO.

In addition to above-mentioned desorbing agents, distilled water (DI H_2_O) was also applied to desorb Cr^6+^, Fe^3+^ and Ni^2+^ ions from spent CTS/PVA/Zeo membrane-based NEMs [104], and its desorption efficiencies of Cr^6+^, Fe^3+^, and Ni^2+^ ions were 99.0%, 92.0% and 96.0% with the efficient desorption, respectively. The results suggested that the adsorption capacity of the membrane was unchanged after five cycles. In particular, 0.03 M H_2_SO_4_ solution was selected to desorb Cu^2+^ ion from CTS membrane-based PMs [130], because this desorbing agent could offer a large number of H^+^ to replace Cu^2+^ ion as well as chemically friendly to CTS, the result suggested that the regeneration and reuse of this membrane were easily obtained in 5 cycles. In another study, the desorption process of Cu^2+^, Zn^2+^, Cd^2+^, and Ni^2+^ ions was carried out to regenerate with three different desorbing agents including 0.05 M EDTA, 0.05 M DTPA, and 0.05 M Ca(NO_3_)_2_ aqueous solutions from CPBC membrane-based PCMs [65]. For the electrolyte desorbing agent, Ca(NO_3_)_2_ showed ineffectiveness in metal ions adsorption removal from the composite materials. In contrast, the EDTA and DTPA aqueous solutions could desorb up to 90.0% of the adsorbed metal ions owing to their chelating functional groups with metal ions. In particular, the Cu^2+^ desorption efficiency was lower that of other metal ions due to the formation of multinuclear complexes with the composite material, as well as the desorption succession of studied metal ions accorded to their relative stability of the ligand complexes.

Overall, the adsorption capacity of metal ions on the AMs was almost reduced with rising the number of the recycling cycle during repeated adsorption/desorption processes because of the loss of active sites. However, depending on the study's aims, the regeneration and recycling of the adsorbent are truly indispensable to be applied widely in industries with practical applications. Therefore, to become a potential adsorbent, in addition to the high adsorption capacity, its desorption efficiency also needs to be better to improve the adsorption efficiency of the AMs and reduce the cost.

## Influence of morphological structures of AMs

Generally, an affinity complex in AMs is formed leading to be slower in the rate-limiting mass transport process. Besides, AMs with large surface areas and intra-particle diffusion result in short residence times, low backpressures, and large volumetric capacity in the large-scale. Hence, the morphological structure of AMs is concerned as a significantly important factor that impacts to heavy metal ions removal performance in the adsorption process. In addition to the specific adsorption groups on AMs, the morphology of AMs is also needed to concern the removal efficiency for heavy metal ions from aqueous solutions. Theoretically, the thin membrane with greater pore size is favorable for the operation of high flux and low pressure as required by AMs.

In the case of PMs, the morphological structure of CTS membrane was porosity structure, the porosity ones of the crosslinked CTS membranes would be reduced after crosslinking [50]. It related to rising the hydrophobicity of the membranes because the alkyl groups were added by the crosslinking reactions. Besides, GLA was used to crosslink PVA nanofibers membrane since the non-crosslinked PVA nanofibers could be dissolved in water [85]. CTS/PEG membrane was a pore microstructure with dense and uniform one, the size of this pore structure would be bigger for the higher content of PEG [48]. In another study, CTS/PVA membrane was created with a dense structure and no porosity through casting evaporation method [54]. Thus, it is truly needed to modify the morphological structure of this membrane by the addition of hydrophilic PEI into the casting solution, this modified membrane surface would be less dense due to the improvements of the hydrophilicity of the membrane. However, increasing the amount of PEI led to increasing the thickness and density of the membrane, as well as decreasing the porosity of the sublayer.

Furthermore, the use of inorganic solid particles is seen as a common method to create membrane pores. In contrast, its disadvantage is the deposition of these solid particles in casting solutions, which lead to forming the asymmetric pore structure of the fabricated membranes. Hence, to avoid this defect, the viscosity of the casting solution should be controlled carefully through polymer concentration to hinder or slow down the deposition of these particles. In particular, Wang et al. fabricated CTS membrane adsorber with lots of pores that appeared in both the top and the bottom surfaces of this membrane adsorber [130]. Besides, the pores structure of this CTS membrane was interconnected and symmetric led to being better dynamic adsorption through filtration. The case of NEMs, both the PAN and CTS layers all were bead-free nanofibers, use of metal oxides (ZnO and TiO_2_) nanoparticles into PAN/CTS nanofibers membrane made the electrospinning solution more viscous; therefore, the surface of PAN/CTS/metal oxides nanoparticles (PAN/CTS/MO) membrane be rougher [131]. These changes in the surface morphological structure of AMs will influence to the heavy metal ions adsorption efficiency from aqueous solution.

In the case of PCMs, PCMs with porous structure and high surface area are almost applied for catalytic and absorptive applications [57], as well as a large area is truly required to interact with reactants. As known, the surface of raw bentonite was known to be a smooth one with irregular shapes and aggregated together via intermolecular forces, which led to considering recently in the modification of bentonite surface. For example, Liu et al. carried out successfully the surface modification of PANI on bentonite surface (PANI/bentonite, PANlB) [66], suggesting that morphological property of PANlB was the thin PANI layer encapsulated the structures of plate-like bentonite. Besides, the morphological property of CPBC (CTS-grafted-PAA-bentonite composite) membrane attained a porous structure with an extensive unfolded 3D network [65], their crosslinking reaction led to rearranging polymer chain with clay particles. However, it also depends on the amount ratio of components and the preparation process (e.g.: manual compaction, the firing process, etc.) to be attained the surface morphological structures appropriately. Specifically, Ali et al. investigated the morphological characterization of clay/sawdust mixture-based PCMs before and after the filtration process [57], which was reported through SEM results (Fig. 6). The removal efficiency for Pb^2+^, Cu^2+^, and Cd^2+^ ions from water achieved with high values (> 99.0%) through chemical analysis results. In general, these PCMs with different weights of sawdust (0.2–5.0% wt.) were the structures with rough surfaces before the filtration process (Fig. 6a–f), mainly due to their irregular shapes, sizes, and distribution in the pores. Moreover, the depressions and microcracks were also appeared on the membrane surface to be attributed the manual compaction and deformation during the ceramics firing process, respectively. These PCMs generally contain –OH groups on the pores surface to contact with the heavy metal ions; for instance, the heavy metal ions were adsorbed on the sides of the PCM pores after filtration process (Fig. 6g–i), as well as the heavy metal ions with smaller sizes were still adsorbed on the PCM pores by the adsorptive active sites.

In the case of ENMs, in addition to set-up parameters of the whole electrospinning method, the control of the amount ratio and selection of material is also truly important to prepare promising nanofibers membranes. Especial for PEI/PVA membrane-based ENMs, it is significantly important to select appreciative mass ratios of PEI in the fabrication of PEI/PVA membrane [132]. Additionally, the fabrication of ENMs from cellulose material is seen as a challenge in the electrospinning method mainly due to its insoluble property in various solvents (i.e.: strong intra and inter-molecular hydrogen bondings). To improve this disadvantage, the cellulose material was modified through thiol-functional groups and deacetylation processes to form the TC membrane [98], resulting in that TC nanofibers were attained a uniform structure and a smooth surface with a random-orientation. For CNC/CTS/PVA-SH (cellulose nanocrystals/CTS/PVA nanofiber membranes with thiol-functionalized) membrane-based ENMs, its surface morphological property was changed from a smooth state to a rough one with the increase in an appreciative weight ratio of CNC [97]. The nanofiber's diameter was uniform and regular with the addition of 5.0 wt% CNC, but it is un-uniform with the addition of 20.0 wt% CNC. Also, the morphology of neat PAN membrane-based ENMs was usually damaged or varied by amidoximation treatment leading to be more porous in AOPAN (amidoxime PAN) membrane-ENMs owing to conglutinating together of these nanofibers. Hence, Feng et al. conducted hydrolysis/deacetylation and amidoximation treatments to fabricate PAN/RC (PAN/regenerate cellulose) and AOPAN/RC (AOPAN/regenerate cellulose)-based ENMs. The results suggested that these nanofibers were relatively uniform, as well as their surface morphologies were little different [84]. However, these membranes after Fe^2+^, Cu^2+^, and Cd^2+^ ions adsorption were not much different for their morphology structure. As such, the combination of RC into these EMNs made AOPAN/RC membrane more elevated morphological stability comparing to the amidoximation treatment, as well as the advantages of ENMs could be retained.

In the case of GO-based NEMs, the surface morphology of GO membrane was few wrinkle ones with few-layered GO nanosheets by casting method, as well as there was the appearance of fracture edges on the membrane. However, GO membrane could attain a relatively smooth surface and large lateral dimension by vacuum method [26]. The layers of GO membrane were well ordered due to the directional flow during vacuum filtration. In another study, Sitko et al. conducted the fabrication of GO/cellulose membrane by pressing and non-pressing methods [121], the results suggested that the pressed membranes were stable more than the non-pressed membranes during strongly shaking in the aqueous solution. Besides, a thin layer of the non-pressed membrane was peeled off from the surface of nitrocellulose during strongly shaking in the aqueous solution; however, these membranes could be employed in the filtration process at the high flow-rates as well. Moreover, GO incorporated PSf membrane to develop GO/PSf membrane with a more open spongy structure leading to higher permeate flux [120]. Overall, depending on the purpose of the studies, the kind of materials, weight ratio of compounds, and fabrication methods are selected accordingly to achieve the desired results for the morphological property of AMs, which are to improve the adsorption efficiency for heavy metal ions from water.

## Influence of chemical structures of AMs

In addition to the morphological structure of AMs, their chemical structure plays an equally important role as well. Various types of materials include CTS/PEG, nCTS, ECH-cCTS, GLA-cCTS, nCTS, ECH-cCTS, GLA-cCTS, CTS, PVT-*co*-PAN, PVA/PEI, CTS/PVA/PEI, CA/PEI [48, 50, 52–55, 129, 130], which were employed as PMs to remove heavy metal ions by the adsorption process (Table 2). Among the polymer materials, CTS and nCTS (acidic) were considered the most promising materials for the adsorption removal of heavy metal ions based on higher adsorption capacity (800–1400 mg g^−1^, Table 2) for Cr^6+^ ion. Indeed, from the unit cell structure of the CTS (Table 3), it is easily understood that the multiple groups containing oxygen and -NH_2_ are available in the CTS structure [46, 47, 119]. All of these groups work as anchoring groups of heavy metal during the adsorption process. Besides, these functional groups help to disperse the materials to the other matrices through interfacial interaction. As a result, the prepared AMs composites incorporation of the CTS, nCTS, and Cellulose are more mechanically stable compared to the other materials matrix, like PVA, PEI, ECH, GLA, GO, AC, etc. The matrix materials have more functional groups (–OH, –NH_2_, –C=O, Cl, or other types of ligand), it is truly beneficial to prepare a high-performance AM membrane. The porosity of the AMs materials could be preserved after the recycling process due to the stable structure. The case of PCMs, CPBC (CTS-grafted-PAA-bentonite composites) [65] showed good adsorption capacity (Table 2) for the removal of Cu^2+^, Zn^2+^, and Cd^2+^ ions. This result is due to the multifunction anchoring groups of CTS and bentonite (source of montmorillonite, which has a lot of oxygen terminal anchoring point of -OH and metal oxides). Bentonite also supports the adsorption removal of Cu^2+^, Zn^2+^, and Cd^2+^ ions at adequate levels in PANOB (PAN/organobentonite) [58] membrane owing to its oxygen terminal anchoring point on -OH and metal oxides.

**Table 3:** Chemical structure of AMs

Materials	3D format structure	2D format structure
PEG		
PVA		
PAN		
PVT		
PEI		
CTS		
Cellulose (or saw dust or wood)		
CA		
PSf		
ECH		
PAA		
GO		
Montmorillonite	Large structure containing Na, Ca, Al, Si, OH, and nH_2_O	Large structure containing: (Na, Ca)_0.33_ (Al Mg)_2_ (Si_4_O_10_) (OH)_2_ nH_2_O
CMC	–	CTS/montmorillonite composite
CCPM	–	Crosslinked CTS/Al_13_ pillared montmorillonite
CPBC	–	CTS grafted PAA-bentonite composite
Fe oxide-CMC	–	Fe_3_O_4_/CTS/montmorillonite complex
Al oxide-CMS	–	Al_2_O_3_/CTS/montmorillonite complex
Bentonite	–	Source of montmorillonite

The multifunctional groups (–OH and –NH_2_) also played an important role in the absorption removal of heavy metals in ENMs. The adsorption capacities of Cd^2+^, Pb^2+^, Cr^6+^, Cu^2+^, and Co^2+^ ions were observed to be high for PAN/CTS [100] and PEI/CTS materials [99]. Interestingly, CTS itself showed lower adsorption capacity when used alone without another matrix for the removal of Ni^2+^ and Cu^2+^ [96]. Generally, CTS is soluble in water, and its stability is comparatively poor. Making stable ENMs with CTS requires other polymer or binding materials. Increasing adsorption required the inclusion of –SH and sulfone (O=S=O shown in Table 3) group-containing polymers in CNC/CTS/PVA-SH [97] and PEI/PES [89]. -SH and sulfone groups work as anchoring groups of the metal ion either by coordination or by electron donation as a ligand. Regenerative cellulose was incorporated in AOPAN [84] to increase the adsorption performance of Fe^2+^, Cu^2+^, and Cd^2+^ ions. Cellulose has a hexagonal structure containing multifunctional oxygen groups like CTS (Table 3), making it a good option for AM technology. However, due to lack of –NH_2_ groups compared to CTS, it may not be as effective as CTS for adsorption. Furthermore, in NEMs, the adsorption performance of the PAN/MO/CTS [100] membrane was enhanced by the hexagonal structure containing multifunctional oxygen groups such as CTS. The Cd^2+^ and Pb^2+^ ions adsorption of PAN/MO/CTS was increased four and two times, respectively, relative to the PAN/MO [100] membrane (Table 3). Similarly, good adsorption performance was also observed for Pb^2+^, Co^2+^, and Ni^2+^ ions in CTS/HAp [123] membrane. It showed an excellent adsorption capacity of Cu^2+^ ion due to the oxygen and -OH multifunctional groups in Zr-MOFs [124].

Overall, the multifunctional groups, such as oxygen (O), nitrogen (N), sulfur (S), and other electron-donating materials, are necessary for the development of high-performance AMs. Therefore, controlling the chemical structure of materials by the electron-donating or coordinating groups is necessary, as they are the driving force for heavy metal ion adsorption in AMs for varying temperature and pH. Furthermore, the porosity of the AMs could be tuned to the desired level by the incorporation of multifunctional groups.

## The outlook of future research

The cost of fabrication is an important factor in the development of AMs. It can be reduced to the desired level by (i) low-cost synthesis process, (ii) reusing the AMs, and (iii) enhancing the removal performance of heavy metal ions by the AMs. To regenerate and reuse the AMs, AMs have to be stable in chemical factors and the performance of the recycling should be near to the first cycles. Therefore, to maintain the adsorption capacity during the repeated use of the adsorbents in wastewater treatment or purification, recycling performance is seen as one of the important factors in the adsorption process. The general techniques for recycling are done by desorbing the heavy metal ions from the spent AMs. The common desorbing agents are NaOH, NaCl, Ca(NO_3_)_2_, HCl, H_2_SO_4_, HNO_3_, EDTA, Na_2_EDTA, and DTPA. Among them, the EDTA, Na_2_EDTA, and DTPA aqueous solutions are employed as strong complexing agents for excellent desorption, but the cost of these desorbing agents are not inexpensive. Thus, to reduce the cost for the recycling process, the inorganic acid/base (HCl, HNO_3_, and NaOH) and NaCl aqueous solutions were the most suggested owing to their inexpensive cost and desorption efficiency. Even though the high performance (~ 96.0–99.0%) of the distilled water (DI H_2_O) as the desorbing agent for Cr^6+^, Fe^3+^, and Ni^2+^ ions from spent CTS/PVA/Zeo membrane-based NEMs [104], but it was not widely applied for the other AMs. If it is applicable with good performance to all of the AMs, it might be a good option. To further improve in the recycling performance, it truly needs a very simple technique that desorbs the heavy metal ions from the AMs without using any salt or acid or base.

The recently advanced adsorbents from nanoparticles with the special features were used as fillers in the membrane to enhance its performance are detailed in the fabrication of the NEMs. Besides, the arrangement in the morphological structure of the nanofibers membrane is also currently more focused owing to its surface area. Furthermore, the selection of pristine materials or polymers is also one of the significant important factors to practice reasonably in the hybridization and combination of materials together attaining the superior adsorptive properties of the adsorbent. On the other hand, to evaluate the quality of the AMs for practical use, the avail oneself of these AMs have to be stable in chemical factor and reusable.

In addition to the importance of the above-mentioned polymeric materials and nanomaterials of AMs, the ceramics materials are concerned with one of the engaging natural material sources owing to its hydrophilic behavior and specific functional groups toward charged metal ions in the adsorptive property. Besides, these ceramics materials display superior physical properties [i.e.: high temperature and pressure], which is one of the promising potential candidates to be further developed as the AMs systems in wastewater treatment or purification. At the same time, the MOFs is also one of the interesting materials to be developed much more in the fabrication of NEMs. Thereby, the ENMs and NEMs are probably seen as one of the advanced technologies in the fabrication of AMs to be aimed for the enhancement of the heavy metal ions adsorption capacity.

In general, the adsorption volume of metal ions on the AMs was almost decreased with increasing the number of the recycling cycle numbers during repeated adsorption/desorption processes because of the loss of active sites. However, depending on the study's aims, the regeneration and recycling of the adsorbent are a truly crucial factor to be applied extensively in industries with practical applications. Therefore, future research will be needed for smart AMs developments by applying the click chemistry in which deactivated pore of AMs can be activated without decreasing surface area and surface functional groups, regeneration, and reuse of AMs can be done without acid or base or salt without hampering recycles performance.

## Conclusions

The development of AMs (PMs, PCMs, ENMs, and NEMs) and the recent progress in advanced adsorbents (nanoparticles) are summarized. The above-mentioned investigations indicated that the prepared AMs could effectively remove heavy metal ions from water as well as their regeneration and reuse ability, due to their exclusive morphological surface and chemical structure features. For further improvement, need advanced research on the recycling of the AMs for large number cycles with excellent adsorption and desorption performance without using any expensive desorbing chemicals. To preserve the surface area and active site of AMs, need fabrication process development and also utilization of multiligand or multifunctional materials, which works as heavy metal ions adsorbing antenna in various environments. Although the incorporation of nanoparticles in the nanofibers membrane supported both larger surface area and porosity as well as high adsorption capacity, the expansion and improvement in the fabrication of AMs will be required to create promising AMs with environmental friendliness and inexpensiveness.

## Data Availability

Not applicable.

## Abbreviations

AM(s): Adsorptive membrane(s)
PM(s): Polymeric membrane(s)
PCM(s): Polymer-ceramic membrane(s)
ENM(s): Electrospinning nanofiber membrane(s)
NEM(s): Nano-enhanced membrane(s)
(n)CTS: (Natural) chitosan
PEG: Poly(ethylene glycol)
ECH: Epichlorohydrin
GLA: Glutaraldehyde
PEI: Poly(ethyleneimine)
PVA: Poly(vinyl alcohol)
CA: Cellulose acetate
PAN: Ploy(acrylonitrile)
PAA: Poly(acrylic acid)
PMAA: Poly(methacrylic acid)
EDTA: Ethylenedinitrilo tetraacetic acid
AOPAN: Amidoxime polyacrylonitrile
RC: Regenerate cellulose
PEO: Poly(ethylene oxide)
PT: Permutit
CNT(s): Carbon nanotube(s)
AC: Activated carbon
PSf: Polysulfone
GO: Graphene oxide
CCGO: Magnetic cyclodextrin—CTS/GO
pHEMA: Poly(hydroxyethylmethacrylate)
HAp: Hydroxyapatite
MOF(s): Metal–organic framework(s)
ECH-cCTS: ECH crosslinked CTS
GLA-cCTS: GLA crosslinked CTS
PVT: Poly(vinyl tetrazole)
PANOB: PAN/organobentonite
CMC: CTS/montmorillonite composite
Al oxide-CMC: Al_2_O_3_/CTS/montmorillonite composite
Fe oxide-CMC: Fe_3_O_4_/CTS/montmorillonite composite
Fe oxide-PE_8_M: Fe_3_O_4_/PEI800/montmorillonite
Fe oxide-PE_25_M: Fe_3_O_4_/PEI2500/montmorillonite
CPBC: CTS-grafted-PAA-bentonite composites
TC: Thiol-functionalized cellulose
PANlB: Poly(aniline) modified bentonite
CCPM: Crosslinked CTS/Al_13_-pillared montmorillonite
AgNPs-St-PEG-AcANCH: AgNPs-base starch/PEG-PAA nanocomposite hydrogel
MO: Metal oxide
Zeo: Zeolite
DI H_2_O: Distilled water
HCl: Hydrochloric acid
HNO_3_: Nitric acid
NaOH: Sodium hydroxide
NaCl: Sodium chloride
EDTA: Ethylenediaminetetraacetic acid
Na_2_EDTA: EDTA disodium salt
H_2_SO_4_: Sulfuric acid
Ca(NO_3_)_2_: Calcium nitrate
DTPA: Diethylenetriaminepentaacetic acid
